# Improving AlphaFold2 Performance in Virtual Screens Targeting GPCRs by Enhancing Binding-Site Conformational Sampling

**DOI:** 10.1021/acs.jcim.6c00034

**Published:** 2026-05-04

**Authors:** Núria Mitjavila-Domènech, Alejandro Díaz-Holguín, Huabin Hu, Nour Aldin Kahlous, Israel Cabeza de Vaca, Björn Wallner, Jens Carlsson

**Affiliations:** † Science for Life Laboratory, Department of Cell and Molecular Biology, 8097 Uppsala University, BMC Box 596., SE-751 24 Uppsala, Sweden; ‡ Division of Bioinformatics, Department of Physics, Chemistry and Biology, Linköping University, 581 83 Linköping, Sweden

## Abstract

Artificial intelligence has transformed protein structure prediction, with AlphaFold2 (AF2) generating models with near-experimental accuracy. However, as AF2 was trained to generate a single structural model, this method does not capture the conformational flexibility of proteins, limiting its utility for structure-based drug design. Here, we explore strategies to generate diverse ensembles of binding-site models suitable for structure-based virtual screening against G protein-coupled receptors (GPCRs), a major class of drug targets. Our AFsample2T approach uses multiple sequence alignment column masking in the receptor binding site to reduce coevolutionary signals, leading to greater structural heterogeneity in the generated models. We demonstrate that ensembles of AFsample2T models capture multiple relevant binding-site conformations and reproduce experimentally observed conformational variability. Evaluation of AF2-based models in structure-based virtual screening using docking of actives and decoys shows that considering ensembles of diverse binding-site models substantially improves ligand enrichment. Our results provide guidelines for using AF2-based models in structure-based ligand discovery for GPCRs, and the AFsample2T approach can readily be applied to other protein classes.

## Graphical abstract

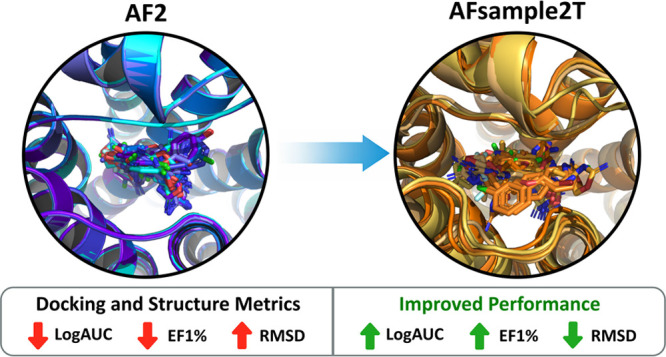

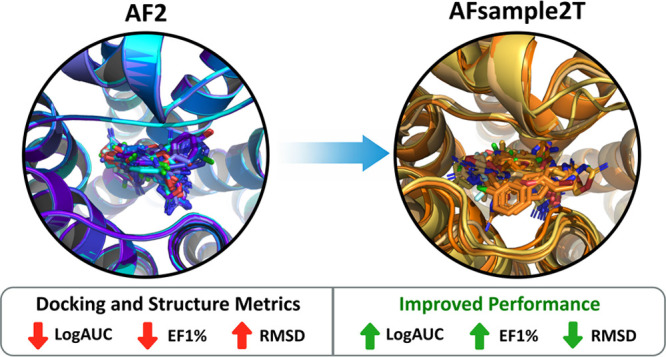

## Introduction

Advances in artificial intelligence (AI) have transformed the field of protein structure prediction. Deep-learning methods such as AlphaFold2 (AF2) and RosettaFold can predict protein structures with near-experimental accuracy and outperformed traditional techniques in community-wide assessments.^1–6^ One potential AF2 application is in the area of structure-based drug design, which relies on access to high-resolution models of proteins. Detailed structural insights into the architecture of a binding site enable virtual screening of large chemical libraries to identify starting points for drug discovery and can guide compound optimization.^7^ In particular, accurate structure prediction methods can provide models for proteins that are difficult to characterize experimentally, such as membrane proteins.^8–10^

Since AF2 was trained to predict static structures, the method may not capture multiple conformational states or the structural heterogeneity of proteins.^11–13^ This is a major limitation that affects the utility of AF2 for structure-based drug design. First, several important classes of proteins transition between functional states, such as the active and inactive conformations of receptors or the inward- and outward-facing conformations of transporters. Many drugs act by stabilizing a specific state, but AF2 models generally capture only one of the relevant conformations. To address this limitation, strategies to increase sampling of alternative conformational states have been developed by modifying AF2, such as using shallow or clustered multiple sequence alignments (MSA).^14–22^ Second, modeling of protein-drug interactions is sensitive to small variations in the binding pocket shape, and even minor errors in the side-chain conformations of AF2 models can be detrimental to the accuracy of molecular docking methods.^23,24^ Despite the high overall accuracy of AF2, the lack of local structural heterogeneity in predicted structures limits the ability of the method to capture the pocket conformations relevant for drug binding.^25,26^ This explains the poor performance of AF2 compared to experimental structures in virtual screening benchmarks, indicating a need for further model refinement to enable drug discovery applications.^27,28^

In this work, we explore strategies to improve the performance of AF2-based models in virtual screening by developing techniques to generate structurally heterogeneous ensembles of binding-site models. We focus on the group of G protein-coupled receptors (GPCRs), a large protein family that shares a conserved fold and are important drug targets.^29–32^ We present the AFsample2T approach, which achieves increased binding-site plasticity by performing targeted conformational sampling using MSA column masking.^33^ Ensembles of AFsample2T models capture multiple relevant binding-site conformations with backbone and side-chain variability similar to experimental structures, representing an improvement over the default AF2 implementation. Predicted and experimental GPCR structures were also evaluated using molecular docking calculations to identify models suitable for virtual screening. We demonstrate that AF2-based models with improved virtual screening performance can be identified by enhancing conformational sampling.

## Results

### Increasing Binding-Site Conformational Sampling by MSA Masking

The first objective of our work was to enhance the conformational diversity of ensembles generated by AF2 to better reflect the structural variation observed experimentally. By masking columns in the MSA, the input for AF2, the coevolutionary information that put constraints on the predicted structures can be attenuated (Figure 1A). Masking can be applied to the entire protein sequence or targeted to specific regions. This approach has been demonstrated to increase the structural diversity of the models and predict alternative states of proteins.^33^ We focused on class A GPCRs, which share a conserved tertiary structure composed of seven transmembrane (TM) helices connected by extracellular and intracellular loops. The orthosteric binding site of class A GPCRs is located in the extracellular-facing region of the protein, and the architecture of this pocket varies depending on the nature of the native ligand (Figure 1B).^34^ As our focus was to increase sampling in the orthosteric binding site rather than exploring large conformational changes in the receptor, we used a targeted sampling approach. Based on determined GPCR structures, the MSA masking region was defined as the extracellular TM region and the part of extracellular loop 2 (EL2) that interacts with orthosteric ligands in many receptors (Figure 1B). In addition to the local structural variation in the binding site, GPCRs can adopt two major conformations: the active and inactive states.^35^ The inactive receptor state was predicted using only the receptor sequence. The active state was modeled by using the sequences of the receptor and heterotrimeric G protein, which binds to an intracellular pocket and leads to large conformational changes in TM6 of the receptor.^35^ In agreement with the work of Chiesa et al.,^17^ comparison of the AF2 models with experimentally determined structures of active and inactive states of four receptors confirmed that this approach reliably captures the characteristic conformations of TM6 (Table S1).

**Figure 1:**
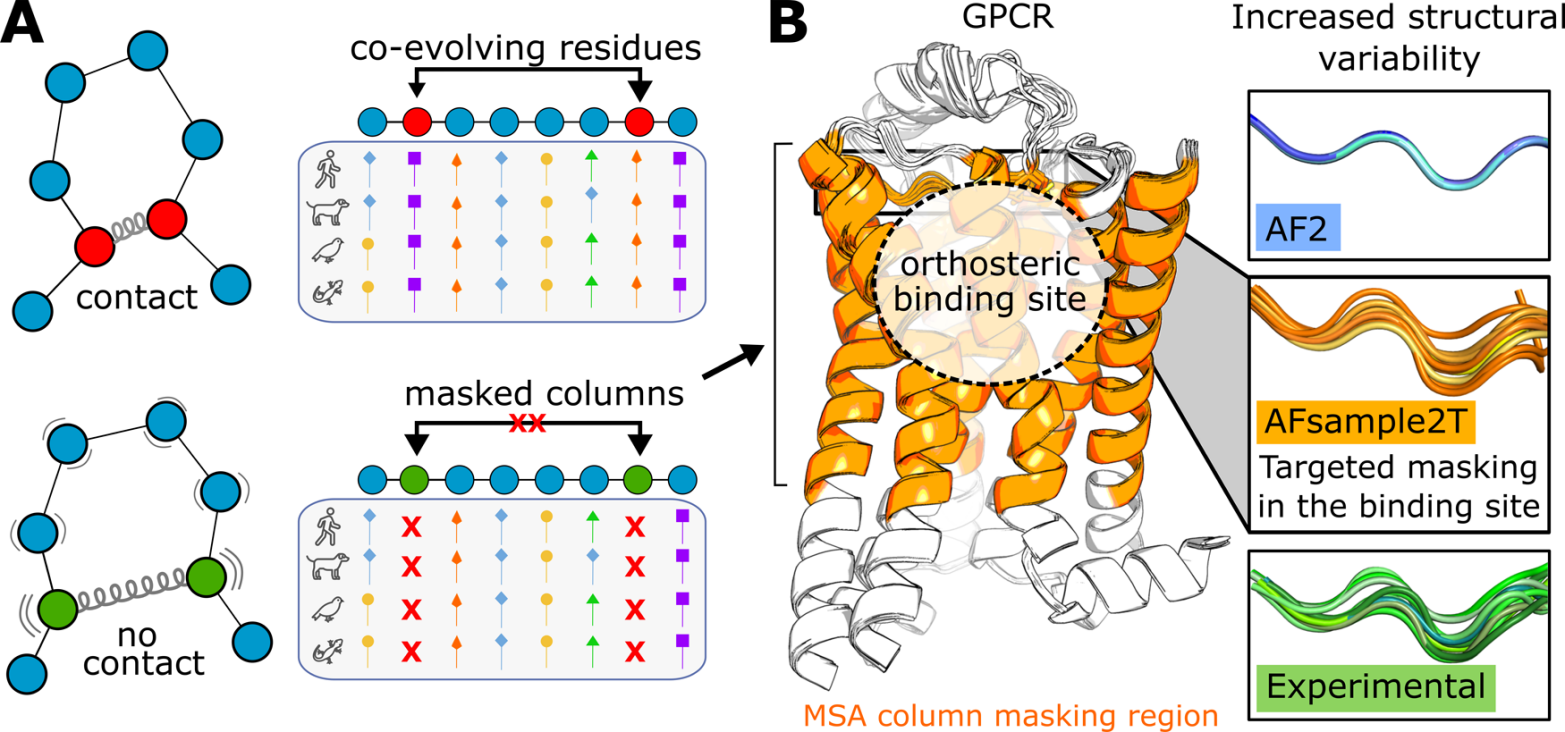
Workflow for enhancing GPCR structural diversity using the AFsample2T approach. (A) Introduction of MSA column masking reduces the amount of coevolutionary information provided to AF2, leading to greater structural variability in the generated models. (B) The selected MSA column masking region for class A GPCRs, which includes the TM region forming the orthosteric binding site and part of EL2. The receptor is shown as a cartoon, and the MSA column masking region is colored orange. The ensemble of models generated with MSA column masking (AFsample2T; orange) exhibits higher structural variability than the default AF2 (blue), aligning more closely with the protein flexibility observed experimentally (green).

### MSA Column Masking Can Improve Binding-Site Accuracy

To evaluate the effect of MSA column masking on the binding-site models, we generated AF2 models without masking (the default) and with targeted masking probabilities of 10%, 20%, 30%, or 50% applied to residues forming the orthosteric binding site (Figure 1B). For comparison, we also generated models using the standard AFsample2 technique, which applies column masking (15%) to the full MSA. Column masking selectively hides positions in the MSA during model inference, thereby reducing coevolutionary signals at those positions and allowing AF2 to explore alternative backbone and side-chain arrangements. The masking probability level controls the degree of binding-site variability. A higher masking level introduces more extensive sampling by releasing evolutionary restraints. In the AFsample2T and AFsample2 calculations, we also enabled dropout at the inference stage, which has been demonstrated to increase conformational diversity.^33^ For each masking level, we evaluated the structural accuracy of models for a benchmarking set of 10 class A GPCRs. This set included receptors that recognize diverse compounds, including peptides and small molecules, with orthosteric sites varying in shape, size, and polarity. A total of 119 experimentally determined crystal or cryo-electron microscopy (cryo-EM) structures of these receptors were available, and the number of structures for each protein ranged from 4 to 35 (Table S2). A majority of the structures were determined of the receptors in complex with ligands, but the set also included apo structures of three receptors (5-HT_1A_, H_1_, and M_4_). Low-resolution structures, duplicates of the same ligand–receptor complex, and receptors containing binding-site mutations were excluded (Table S2). After this step, 61 structures were retained for benchmarking of binding-site accuracy. A set of 1,000 models per receptor was generated and accuracy was assessed by calculating the fraction of experimental binding-site structures reproduced within root-mean-square deviation (RMSD) thresholds ranging from 1 to 2 Å (RMSD ≤ threshold). For each masking level, the 1,000 models were aligned to the corresponding experimental structures, and the symmetry-aware side-chain RMSD values were computed for residues within 5 Å of the ligands. The accuracy of the different masking levels was then analyzed over a range of different RMSD thresholds and quantified by calculating the area under the curve (AUC) (Figure 2A).

**Figure 2:**
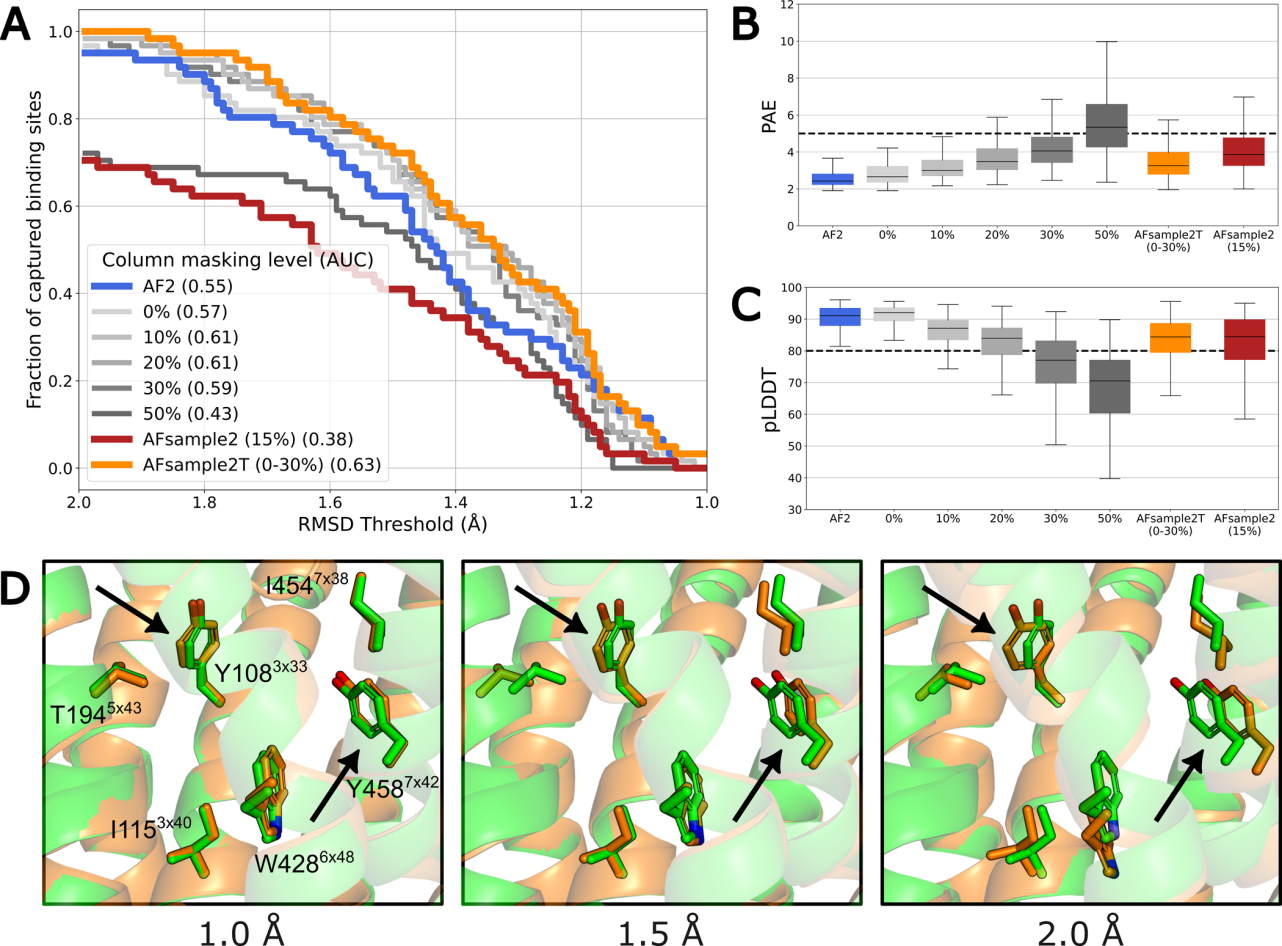
Structural accuracy of binding-site models for different column-masking levels. (A) Fraction of experimental binding-site structures captured by the receptor models over a range of binding-site RMSD thresholds. For each threshold, all structures with RMSDs ≤ the threshold are included. Introducing dropout (0%) and increasing (10%, 20% and 30%) the masking level can improve structural accuracy compared to the default AF2, but performance is reduced at the highest level (50%). The AFsample2T (0–30%) ensemble combines models generated with masking levels of 0%, 10%, 20%, and 30% with dropout enabled. The standard AFsample2 technique uses a masking level of 15% applied to the full MSA. The AF2 models were generated without dropout. (B–C) Increased masking levels lead to a reduction of model confidence: (B) Boxplots representing PAE score distributions of generated models, with a value of 5 used as a reference for acceptable accuracy (dashed line). (C) Boxplots showing pLDDT score distributions of generated models, with a value of 80 used as a reference for acceptable confidence (dashed line). AFsample2T achieves higher model accuracy than AF2 while maintaining acceptable model confidence. (D) Binding-site models with different levels of accuracy (RMSDs of approximately 1.0, 1.5, and 2.0 Å). The predicted and experimentally determined structures of the H_1_ histamine receptor are shown as cartoons with side chains in sticks (orange and green, respectively). Black arrows indicate two residues showing large structural variation in the models.

Receptor models generated using the default AF2 yielded an AUC of 0.54 (Figure 2A). Introducing dropout (0% masking) enhanced structural accuracy (AUC = 0.57). Adding column masking probabilities of 10%, 20%, and 30% further improved structural accuracy, resulting in AUC values of 0.61, 0.61 and 0.59, respectively. However, at the highest probability level evaluated (50%, AUC = 0.43), predictions of the secondary structure forming the binding site deteriorated, leading to large errors in the models (Figures 2A and S1). Notably, receptor models generated using the standard AFsample2 approach, which applies column masking to the full MSA, yielded the lowest binding-site accuracy (AUC = 0.38), highlighting the effectiveness of targeted sampling. As column masking reduces the amount of sequence information used in the structure prediction, the model confidence can be expected to decrease. We analyzed the predicted Local Distance Difference Test (pLDDT) and the Predicted Aligned Error (PAE) scores, which are the main confidence metrics calculated by AF2^1^. Consistent with expectations, higher MSA masking levels led to a decline in model confidence, as reflected by increasing PAE values and decreasing pLDDT scores. The median pLDDT values for 10%, 20%, 30%, and 50% column masking were 87.1, 83.9, 77.1, and 70.6, respectively (Figure 2B,C). These results indicated that moderate MSA masking (10–30%) enhances binding-site structural accuracy without substantially compromising model confidence, whereas extensive (50%) or global masking (AFsample2) leads to large reductions of model accuracy (Figure 2A-C).

To further optimize prediction accuracy, we combined sets of models generated at different masking levels, which we refer to as the AFsample2T ensemble. A combination of 250 models each from masking levels of 0%, 10%, 20%, and 30% resulted in the highest structural accuracy of the evaluated ensembles (AUC = 0.63, Figure 2A) and maintained high model confidence (median pLDDT = 84.4) (Figure 2B,C). At a binding-site RMSD threshold of 1.5 Å, the AFsample2T ensemble captured 73.8% of the GPCR binding sites, which can be compared to 60.7% for AF2. The enhanced performance of the AFsample2T ensemble between RMSD thresholds of 1.15 and 1.30 is due to more accurate modeling of μ-opioid receptor structures that AF2 failed to capture. In the case of the μ-opioid receptor, AF2 generated relatively collapsed binding pockets compared to those in the experimentally determined structures (Table S3). The binding site volumes in the AFsample2T ensemble were more consistent with experimentally determined structures, highlighting the benefit of using column masking to increase conformational diversity. Analysis of the predicted structures with different binding-site RMSD levels (1.0–2.0 Å) showed that improved models exhibited more accurate predictions of both the backbone and side-chain conformations (Figure 2D).

### AFsample2T Models Exhibit Binding-Site Plasticity Consistent with Experimental Structures

To evaluate the effect of targeted MSA column masking on binding-site plasticity, we calculated the per-residue side-chain and backbone root-mean-square fluctuation (RMSF) values of models generated by AF2, AFsample2T, and the 119 experimentally determined GPCR structures (Figures 3 and S2,S3). The experimental structures exhibited a median side-chain RMSF of 0.58 Å, reflecting the plasticity of the orthosteric site of GPCRs. In contrast, the AF2 models had a median side-chain RMSF value of 0.15 Å, indicating very limited conformational sampling. The AFsample2T ensemble exhibited greater structural variability in the orthosteric site, resulting in a median side-chain RMSF of 0.45 Å. A similar trend emerged if RMSF was calculated using only binding-site backbone atoms. AF2 showed limited conformational sampling (median RMSF = 0.10 Å), while the AFsample2T ensemble and experimentally determined structures exhibited increased variability (median RMSF values of 0.28 and 0.30 Å, respectively). The per-residue binding-site RMSF values from the AFsample2T models and experimental data exhibited a weak correlation (*r* = 0.20–0.40) for the 10 GPCRs (Figure S4). This result may be due to the fact that a small number of structures are available in each case, which will not accurately reflect the conformational heterogeneity of the receptors. Among the GPCRs with the largest number of available structures, a moderate correlation (*r* = 0.41–0.86) for the per-residue binding-site RMSF values was obtained for the D_1_ dopamine receptor and trace amine-associated receptor 1 (TAAR1) (Figure S5). The enhanced conformational sampling was particularly evident in the EL2 region, which often exhibits considerable variability between GPCR structures determined in complex with different ligands. Comparing the 13 available structures of TAAR1 with models from the AF2-generated ensembles showed that AF2 generated nearly identical EL2 conformations, whereas models from the AFsample2T ensemble captured the conformational heterogeneity observed experimentally (Figure 4). The same behavior was observed for the remaining receptors when comparing an equal number of experimental and AF2-based models (Figures S6 and S7).

**Figure 3:**
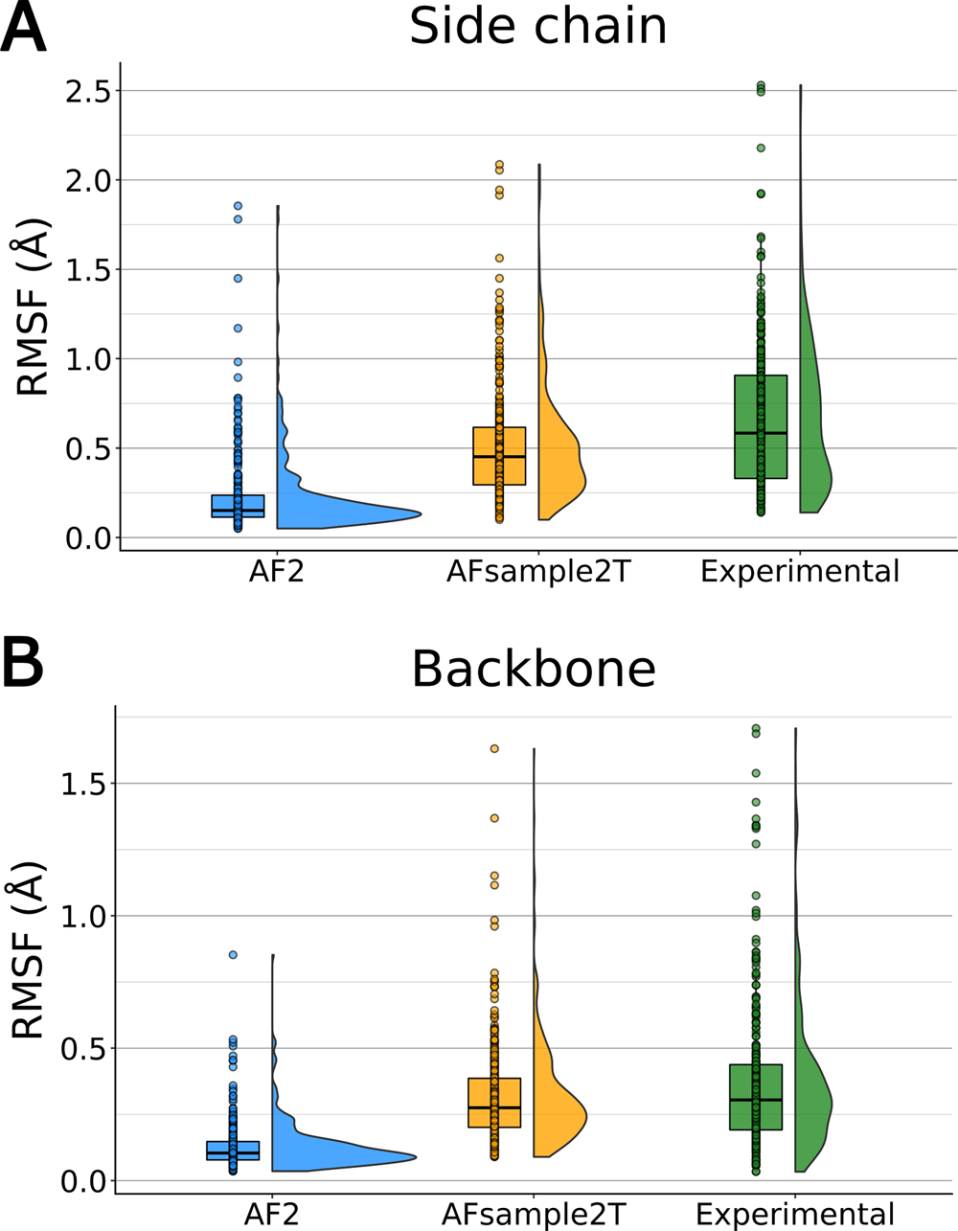
Per-residue RMSF distributions of predicted and experimentally determined structures. Boxplots representing RMSF values for binding-site (A) side-chain and (B) backbone residues of AF2 models, the AFsample2T ensemble, and experimental structures for the 10 GPCRs. The AF2 models show limited structural variability. The AFsample2T ensemble exhibits increased conformational variability and more closely matches the plasticity observed in experimentally determined structures.

**Figure 4:**
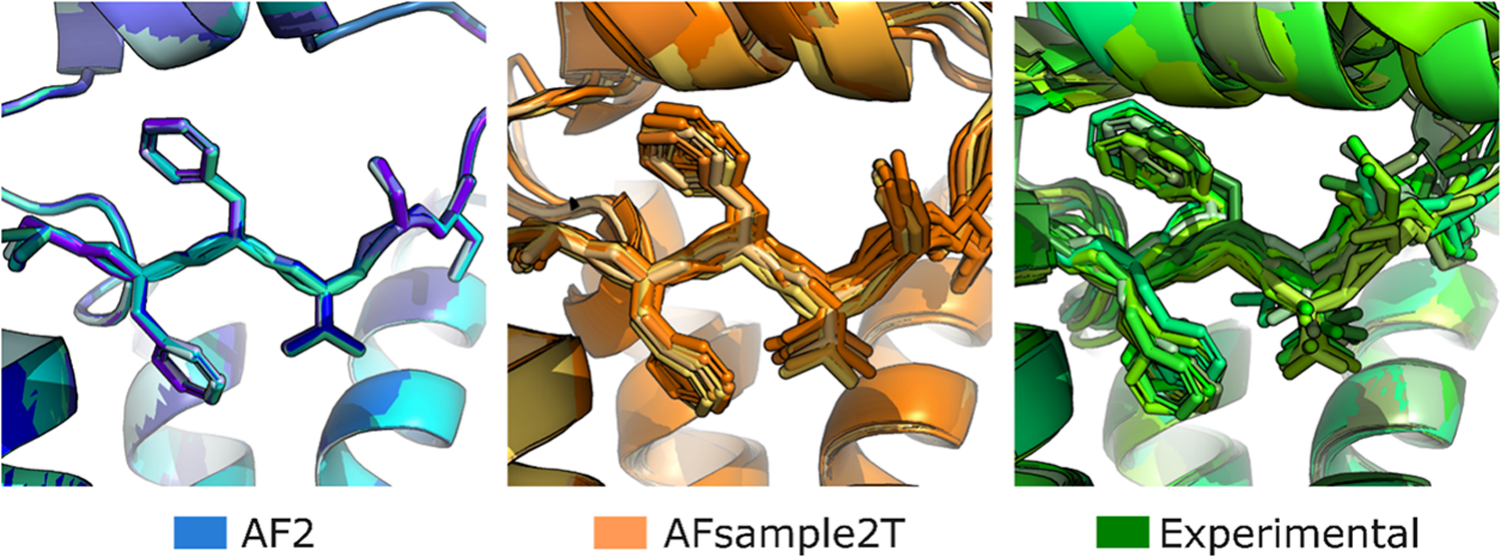
Comparison of TAAR1 models and experimental structures. The EL2 region of TAAR1 from the ensemble of AF2 models (13 randomly selected structures, blue) shows very limited structural variability. Models from the AFsample2T ensemble (13 randomly selected structures, orange) and experimental structures (all 13 structures available in the PDB, green) show larger and comparable structural variability in the same region.

Previous studies have indicated that AF2 generates narrow, collapsed binding sites with side-chain conformations that clash with the ligand binding modes observed in experimental structures.^36–39^ To further evaluate the effect of MSA masking on the orthosteric site, we calculated binding-site volumes for experimental structures and models generated with AF2 and AFsample2T (Table S3). The AFsample2T ensemble exhibited larger binding-site volumes than the AF2 models in 8 out of 10 cases (mean values of 218 and 209 Å^3^ for the 10 receptors, respectively), and the pocket sizes were closer to those in the experimental structures (mean pocket volume = 256 Å^3^). In addition, comparison of the top 1% largest volumes in the ensembles of AF2-based models showed that AFsample2T sampled substantially more expanded pockets (mean volumes of 272 and 389 Å^3^, respectively).

### Virtual Screening Performance of AF2-Based Models and Experimental Structures

Molecular docking screens of known ligands and decoys were carried out to further evaluate AF2-based models and experimental structures. For each of the 10 GPCRs, sets of 1,000 models were generated with AF2 and AFsample2T using 0–30% masking. To ensure that both functional states of the receptors (i.e., active and inactive conformations) were represented, models were generated in both the presence and absence of heterotrimeric G protein (Table S4). The AF2-based models and the 119 available GPCR structures (Table S2) were prepared for docking calculations with DOCK3.8. Sets of ligands from the ChEMBL database (52–202 compounds per receptor) and property-matched decoys were generated for each receptor (Table S5).^40^ Each compound was then docked to the orthosteric binding site with the receptor structure held rigid. Successfully docked compounds were scored using a physics-based scoring function and ranked by their predicted binding energies for each receptor structure. In total, over 240 trillion complexes were predicted and scored to compare the virtual screening performance of AF2, AFsample2T, and experimentally determined structures.^41^ Ligand enrichment over decoys was evaluated using receiver operating characteristic (ROC) curves. For each receptor structure, the logarithm of the area under the curve (LogAUC) and the ROC-based enrichment factor at 1% (EF1%) were calculated.^41^ The EF1% and LogAUC metrics favor early enrichment, which is desirable in virtual screening applications. The adjusted LogAUC (aLogAUC) is obtained by subtracting the area corresponding to random enrichment, and positive values indicate better-than-random discrimination between ligands and decoys.^40,41^ For each GPCR, the median and maximum EF1% and aLogAUC values were calculated based on the 1,000 models. These values were then averaged across all receptors to evaluate the performance of the models and experimental structures. Analysis of results for smaller sets of models (10, 100, 250, and 500) showed that generating ensembles of 100–250 models was typically sufficient to achieve a maximal ligand enrichment comparable to that obtained with 1,000 models (Tables S6 and S7).

By analyzing the median and maximum ligand enrichments of AF2-based and experimental receptor structures, we assessed virtual screening performance across scenarios defined by structure and ligand availability. The maximum ligand enrichment reflects performance when known ligands can guide structure selection through enrichment calculations. The median enrichment reflects the expected performance if an AF2-based or experimental structure is selected at random, a scenario relevant when there are no available ligands. The median ligand enrichment of the experimental structures also represents typical virtual screening performance when only a single structure has been determined. If known ligands are available in such cases, AF2-generated structures can still be evaluated based on enrichment calculations, and a comparison of the top-performing models to the single (or in our case median) experimental structure is relevant. If the median EF1% and aLogAUC were compared, the experimental structures (aLogAUC_median_= 11.2, EF1%_median_= 4.1) outperformed the AF2 models (aLogAUC_median_ = 4.4, EF1%_median_= 1.6) and AFsample2T ensemble (aLogAUC_median_ = 4.2, EF1%_median_ = 1.6) (Figures S8–S10), a result consistent with previous assessments of AF2 models.^23,27,28,37,39,42^ The highest-enriching experimental structures (aLogAU*C*_max_ = 19.6) also outperformed the top-performing 1% of the AFsample2T ensemble (aLogAUC_top1%_ = 12.9) and AF2 (aLogAUC_top1%_ = 10.8) models (Figure 5A). Based on EF1%, the highest-enriching experimental structures (EF1%_max_ = 11.3) and the top models from the AFsample2T ensemble (EF1%_top1%_ = 9.6) yielded more comparable results, whereas the AF2 models performed slightly worse (EF1%_top1%_ = 7.5) (Figures 5B, S10 and Table S8). The top-performing AFsample2T models achieved aLogAUC and EF1% values comparable toand better than those obtained for the experimental structures with median performance, respectively. Notably, the AFsample2T ensemble achieved high and comparable ligand enrichment to the top-performing experimental structures for TAAR1 (aLogAUC values of 32.0 and 27.8, respectively) and the μ-opioid receptor (aLogAUC values of 16.2 and 15.2, respectively). Based on median ligand enrichments (Table 1), the AFsample2T ensemble showed improved results over AF2 in seven cases (5-HT_1A_, D_1_, D_2_, H_1_, M_4_, MT_1_, and TAAR1). The distribution of masking probabilities among the top-performing AFsample2T models highlights the complementary contribution of different levels of MSA column masking (0%, 10%, 20%, and 30%) to the results (Figure 5C).

**Figure 5:**
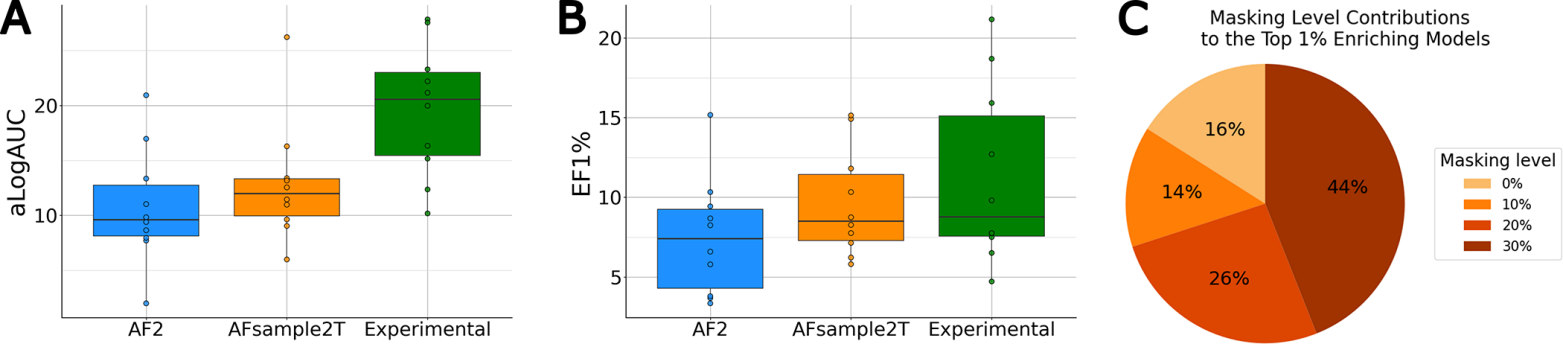
Distribution of aLogAUC and EF1% values across 10 GPCRs. (A) The aLogAUC values for the highest-enriching experimental structure (green) for each receptor and the top 1% of the AF2 (blue) and AFsample2T (orange) models. (B) Distribution of EF1% values for the highest-enriching experimental structures and the top 1% of the AF2 and AFsample2T models. (C) Distribution of models generated with different masking levels among the top 1% of the AFsample2T models.

**Table 1:**
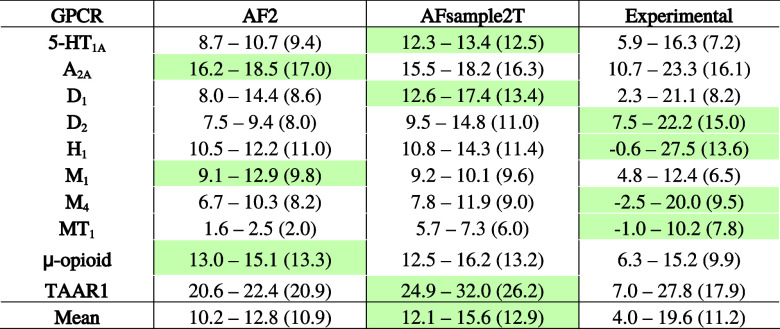
Ligand Enrichment for the 10 GPCRs^a^

a For the AF2 and AFsample2T models, the aLogAUC values correspond to the top 1% performing models (minimum–maximum, with the median in parentheses). For the experimental structures, all the calculated aLogAUC values are included. The best median enrichments are highlighted in green.

## Discussion

Recently developed deep-learning methods are able to predict the three-dimensional structures of proteins with unprecedented accuracy, providing molecular insights that could inform the design of drug candidates. The aim of this study was to improve the ability of AF2 to generate binding-site models relevant for ligand binding and suitable for structure-based virtual screening applications. Our development of the AFsample2T approach for GPCRs yielded three main results. First, targeted MSA column masking increases structural variability in the receptor models, enabling sampling of experimentally observed binding-site conformations not captured by AF2. Second, an AFsample2T ensemble improves sampling of binding-site conformations by 22% over AF2 at an RMSD threshold of 1.5 Å and exhibits pocket characteristics similar to experimental structures. Finally, the ligand enrichment exhibited by AF2-based models in molecular docking calculations can be substantially improved by evaluating an ensemble of predicted structures, and the top-performing AFsample2T models yielded results that were comparable to or better than the average experimental structures. The AFsample2T approach balances structural diversity with accuracy, providing ensembles of high-confidence models while exploring alternative binding-site conformations.

The extraordinary performance of AF2 sparked interest in using predicted structures of therapeutic targets for ligand discovery.^3,43^ Recent studies compared AF2 model performance with that of experimental structures in structure-based ligand design.^28,36,44^ Encouragingly, AF2 models of the 5-HT_2A_ receptor and TAAR1 were successfully used to discover ligands in prospective docking screens, with hit rates comparable to those achieved with experimentally determined structures.^39,45^ Although AF2 generally generates accurate GPCR binding sites, docking to these structures often results in less accurate ligand binding modes compared to experimental structures.^36,38^ Moreover, AF2 models performed substantially worse than experimental structures in retrospective benchmarking studies on diverse drug targets, suggesting that binding-site refinement may be required.^11,23,27,36–38,46,47^ A notable limitation in several previous virtual screening studies is that the performance of a single model and experimental structure of each protein was evaluated. As molecular docking calculations are generally carried out with the protein held rigid, the virtual screening performance is sensitive to small differences in binding-site conformations. The rigid-receptor-approximation affects the performance of both AF2 models and experimental protein structures. However, because widely used benchmarking sets (e.g., DUD-E) have selected experimental structures displaying high ligand enrichment,^40^ comparisons to randomly selected AF2 models may be misleading. In this work, we evaluated ensembles of both predicted and experimental structures, demonstrating that virtual screening performance varies greatly depending on the selected binding-site structure. In agreement with previous studies,^27,28^ we found that AF2 models generally perform worse than experimentally determined structures. However, if our ligand-guided approach is used to select an AFsample2T model, we can identify structures with performance comparable to or better than that of the average experimental structure. Compared to AF2, virtual screening performance is improved by generating ensembles of structures with our AFsample2T approach and, in a few cases, we can achieve ligand enrichment comparable to that of the highest-enriching experimental structures (Table 2). Similar approaches have previously been applied to identify homology models of GPCRs suitable for virtual screening,^48–51^ and the gain in ligand enrichment is comparable to that obtained by combining AF2 models with induced-fit docking.^28^ Together, our results highlight the importance of generating diverse receptor conformations to optimize the virtual screening performance of AF2-based models, which could be further explored by combining the results from screens against multiple models. Docking to sets of AF2-based models has already been applied in prospective virtual screens against TAAR1^47^, but the general application of this approach may require accounting for receptor conformational energies that are challenging to estimate.^52,53^

**Table 2:** Summary of Performance of AF2 and AFsample2T Models in Predictions of GPCR Binding-Site Structures

metric	AF2	AFsample2T
captured binding sites at 1.5 Å (%)	60.7	73.8
median PAE	2.4	3.3
median pLDDT	91.1	84.4
median binding site side-chain RMSF (Å)	0.15	0.45
median binding site backbone RMSF (Å)	0.10	0.28
mean binding-site volume (Å^3^)	209	218
mean top 1% binding-site volume (Å^3^)	272	389
mean aLogAUC of top 1%	10.8	12.9
mean EF1% of top 1%	7.5	9.6

Alternative approaches to AF2-based techniques for obtaining receptor models include molecular dynamics (MD)-based refinement^26^ and the emerging AI-based cofolding methods (e.g., AlphaFold3^56^ and Boltz-2^57^). Compared to AF2-based modeling, MD simulations will be orders of magnitude more computationally demanding and remain technically challenging to perform for membrane proteins and predicted protein structures.^48,54^ Building on advances made by AF2, AF3 and Boltz-2 also enable prediction of structures for receptors in complex with small-molecule ligands. However, performance of the cofolding methods in modeling binding sites remains to be systematically evaluated, particularly for cases lacking close homologues and similar ligands in the training data.^55–59^ A limitation of our protocol is that AF2-based models may not capture relevant receptor conformations if ligand binding leads to substantial induced-fit effects. On one hand, binding site refinement in the presence of a ligand may better capture induced fit than our approach, which is based on conformational selection. On the other hand, induced-fit-based approaches typically focus on a single ligand, which may not generalize to other scaffolds and depend on obtaining an accurate model of the complex. In our approach, model selection is instead guided by evaluating the enrichment of a diverse set of ligands, potentially yielding models relevant to multiple scaffolds.

AFsample2T overcomes a major limitation of the default AF2 method by enhancing the conformational heterogeneity of the generated models in a specific region of the protein. Our approach complements strategies based on either column masking applied to the full MSA or subsampling of the MSA,^14–20,33,60^ both of which are more suitable for capturing large conformational differences, e.g., the active and inactive receptor states. In our approach, the two major conformations of GPCRs are instead modeled explicitly by including the G protein to predict the active state, which has been demonstrated to be an accurate and reliable strategy.^17^ Targeted column masking enables efficient conformational sampling in the binding site region, while maintaining the evolutionary constraints needed to maintain the overall structure of the receptor. By applying a combination of different levels of MSA column masking (0%, 10%, 20% and 30% in equal proportions), our AFsample2T approach generates structural ensembles of high quality by balancing conformational diversity with model confidence (Table 2). In addition, AFsample2T yielded larger binding-site volumes for the majority of evaluated GPCRs, highlighting an advance over AF2 that often predicts collapsed binding-site models.^21,37,38,46,61^ The AFsample2T strategy is flexible, and masking can also be directed toward other regions, such as allosteric sites of GPCRs.^16^ Our approach is particularly well suited to GPCRs due to the conserved fold and binding-site location. However, AFsample2T could readily be applied to other drug target classes, such as kinases,^62,63^ and the source code is openly accessible.

Our results provide guidelines for how to use experimental and predicted structures in molecular docking screening for GPCRs. If several experimental structures of the receptor in complex with ligands are available, one of these is generally preferable for virtual screening applications. This is based on the observation that at least one of the experimental GPCR structures typically achieves virtual screening performance comparable to, or better than, all the generated AFsample2T models. However, it should also be noted that the best AFsample2T models performed better than the average experimental structure for a majority of the GPCRs. If one or only a few experimental structures are available and these exhibit poor ligand enrichment, an AFsample2T model may be a better choice for virtual screening. A limitation of our workflow is that model selection relies on prior knowledge of the binding site location and access to ligands. Virtual screening for receptors with no known ligands remains very challenging, and our results show that model performance will be substantially worse if ligand-guided selection is not possible. Moreover, it should be noted that structures of 4 out of the 10 GPCRs in our benchmarking set were used in the training of AF2, and our approach may therefore not perform equally well for receptors of unknown structure. Prospective studies will be required to further evaluate AFsample2T models, and we suggest the following protocol for virtual screening applications. In our workflow (Figure 6), an AFsample2T ensemble for the relevant receptor state (active or inactive) is first generated to obtain diverse models. In a second step, a control set of actives and decoys is docked to the orthosteric binding site of the models to evaluate virtual screening performance. In the third step, all binding-site models are ranked by ligand enrichment and the top-performing 1% are selected for further analysis. At this point, we recommend detailed examination of the binding site and docked compounds to ensure that the receptor–ligand complexes capture key interactions, in accordance with the best-practices of structure-based virtual screening.^64^ Finally, one or a small set of top-ranked models is selected for prospective virtual screening of large compound libraries. Further development of this GPCR modeling workflow will be focused on the use of templates in AF2 to improve the description of the different receptor states^17,65^ and evaluation of cofolding methods that directly model receptor–ligand complexes.^66,67^

**Figure 6:**
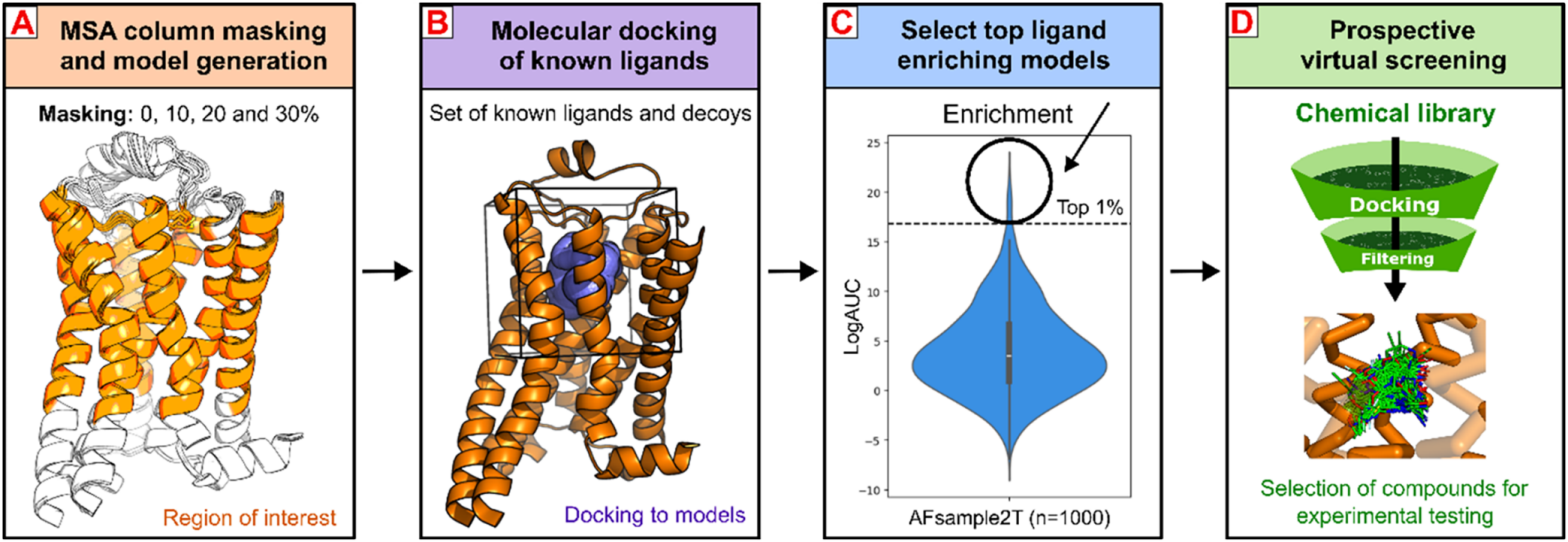
Workflow for the use of AFsample2T in virtual screening applications. Four-step process for generating and applying AFsample2T in prospective virtual screening: (A) Define the binding-site region and generate an AFsample2T ensemble of receptor models using different levels of column masking. We recommend generating an ensemble of at least 250 models. (B) Prepare a set of actives and decoys, and perform molecular docking and enrichment calculations for all models. Sets of at least 10 ligands and 50 decoys per active are typically used in these calculations. (C) Select the top 1% ligand-enriching models for further evaluation. (D) A top-scoring model is used in prospective screens of a large chemical library.

## Materials and Methods

### Generation of AF2-Based Models of GPCRs

Protein structure prediction from sequence was performed using AF2 version 2.3.^1,68^ MSAs were generated using the default AF2 protocol and were modified only if column masking was applied using AFsample2.^33^ The core of the prediction system utilizes a transformer-based model, where attention mechanisms capture relationships within the input data. Specifically, the Evoformer module employs both row-wise and column-wise attention to model relationships between MSA rows (alignments) and columns (residues). This approach enables identification of coevolutionary signals in the MSA that are used to predict the structure with high accuracy. To enhance structural diversity within a GPCR binding site, we used AFsample2T, which introduces targeted MSA column masking specifically applied to residues in the extracellular TM region. We selected residues in the TM region extending up to one helical turn below the PIF motif, a conserved microswitch involved in receptor activation^35^ (P^5x50^, I^3x40^ and F^6x44^; superscripts indicate GPCRdb numbering^69^), as well as residues in the EL2 between the conserved cysteine and TM5.^33^ The specific residues defining these regions are listed in Table S9 for each of the 10 GPCRs. Within these defined ranges, columns in the MSA were randomly selected for masking. Masking probabilities of 0, 10, 20, and 30% were applied to promote the generation of more structurally diverse binding-site models. For each masking probability, 250 structures were generated, with equal numbers of active and inactive receptors. Active models were generated by including the G protein α, β, and γ subunit sequences alongside the GPCR sequence in the AF2-Multimer input.^70^ Inactive models were generated using only the receptor sequence, resulting in structures more closely representing this state. In total, the AFsample2T approach yielded 1,000 models for each receptor: 250 for each masking probability level (0, 10, 20, and 30%). Combined with the 1,000 AF2 models, this resulted in a set of 2,000 models for each receptor. All AF2-based models were generated using the no-template option. Dropout was applied to models generated with masking probabilities (0–30%) and omitted for those generated without masking. The Amber relaxation procedure was applied to all predicted models.^200^

### Evaluation of Binding-Site Models

To evaluate the accuracy of AF2-based models, we calculated binding-site side-chain and backbone RMSDs to the corresponding experimental structures. If the experimental structure represented an active state, the G protein was also included in the models. For each receptor, the binding site was defined as residues within 5 Å of the ligands bound in the determined structures. If several experimental structures of the same receptor–ligand complex were available, the one with the highest resolution was selected. In addition, experimental structures with binding-site mutations were excluded from this analysis (Table S2). Symmetry-aware RMSDs were calculated using in-house scripts. RMSF calculations for experimental structures and AF2-based models were performed using MDTraj.^71^ Binding-site volumes were calculated with Schrödinger’s SiteMap tool^72^ using a reference residue in the orthosteric pocket to define the binding region (Table S3).

### Molecular Docking Calculations

Molecular docking calculations were performed using DOCK3.8.^73^ Docking was carried out to the orthosteric site of 10 different GPCRs. To perform ligand enrichment calculations, a set of actives and decoys was generated. Known ligands of human GPCRs were obtained from ChEMBL^74,75^ (version 33) and activity measurements (K_i_, K_d_, IC_50_, and EC_50_) were converted to their negative logarithmic values. Compounds with p*K*_i_, p*K*_d_, pIC_50_, or pEC_50_ values ≥ 6.0 were retained. These compounds were standardized by salt removal, tautomerization, and charge neutralization using canSARchem^76^ workflows. Additional filtering with RDKit^77^ was performed to retain compounds with less than 25 heavy atoms, no rings larger than seven members, and less than two stereocenters. The remaining ligands were clustered using RDKit Morgan fingerprints (radius = 2) and a 0.5 Tanimoto similarity threshold, resulting in 52–202 ligands per receptor^77^ (Table S5). Property-matched decoys (2,580–10,375 compounds per receptor) were generated using the ZINC20 ^41,78^ library.

The experimentally determined structures were first examined for missing loops or side chains, which were corrected using PyMOL version 3.0.3 or Prime in Maestro. For holo experimental structures, the bound ligand was used to define the binding site. The apo structures used were: 5HT_1A_ (PDB accession code: 7E2X), H_1_ (PDB accession code: 8X5X), and M_4_ (PDB accession code: 6KP6). In these cases, the binding site was defined by aligning each apo structure to the highest-resolution holo structure of the receptor, followed by transferring the ligand coordinates and removing ligand atoms within 2.5 Å of the receptor. For AF2-based models, the same alignment and transfer of ligand atoms procedure was applied. For each AF2-based model and experimental structure, three types of DOCK3.8 grids were generated: van der Waals interactions, electrostatic interactions, and ligand desolvation. For the AF2-derived models, grid parameters and sphere sets were optimized using a subset of 100 models per receptor, and the resulting parameters were then applied to all AF2-based structures. The same docking parameters were optimized individually for all the experimentally determined structures. The receptor grids were optimized through a grid search in which the radii of the thin spheres were systematically varied, starting from 0.1 Å and then from 0.25 Å to 2.0 Å in 0.25 Å increments.^41,64^ One set of thin spheres describes the low protein dielectric and defines the boundary between solute and solvent. A second set of thin spheres determines the magnitude of the ligand desolvation penalty. The thin spheres were optimized to maximize both ligand enrichment and accurate binding modes of known ligands. Partial charge distributions were adjusted to increase the polarity of specific residues, thereby enhancing electrostatic interactions with ligands.^64^ Modified partial charges were applied to two receptors: N^6x55^ in the A_2A_ adenosine receptor, and S^5x43^ and N^6x55^ in the D_1_ dopamine receptor. Docking was performed using a match goal parameter set to 10,000, with a maximum bump of 200 kcal/mol. Successfully docked compounds were ranked using a physics-based scoring function, which includes receptor–ligand van der Waals and electrostatic interactions, corrected for ligand desolvation.^41^ Docking performance was evaluated using ROC curves, with a semilog transformation of the *x*-axis to focus on early ligand enrichment. The LogAUC was calculated, and the aLogAUC was obtained by subtracting the area corresponding to the random curve. The EF1% was calculated as the fraction of ligands identified within the top 1% of the ranked library divided by the fraction of ligands in the entire library.^40,41^

## Supplementary Material



## Data Availability

The scripts used in this study are made available at https://github.com/wallnerlab/AFsample2.

## Notes

Additional tables and figures, includes: summary of experimental GPCR structures; binding-site volumes; ligand control sets; EF1% comparison; binding-site model comparisons; residue fluctuation analyses; and docking enrichment results (PDF)

## Abbreviations

AF: AlphaFold
AUC: area under the curve
aLogAUC: adjusted logarithm of the area under the curve
EF1%: enrichment factor at 1%
EL2: extracellular loop 2
GPCRs: G protein-coupled receptors
LogAUC: logarithm of the area under the curve
MD: molecular dynamics
MSA: multiple sequence alignment
PAE: predicted aligned error
PDB: protein data bank
pLDDT: predicted local distance difference test
RMSD: root-mean-square deviation
RMSF: root-mean-square fluctuation
ROC: receiver operating characteristic
TAAR1: trace amine-associated receptor 1
TM: transmembrane.
