# Supplementary material for: Improving AlphaFold2 Performance in Virtual Screens Targeting GPCRs by Enhancing Binding-Site Conformational Sampling
Source: J Chem Inf Model. 2026 May 4;66(10):6045–56. doi: 10.1021/acs.jcim.6c00034 (PMC13213828; doi:10.1021/acs.jcim.6c00034)
Supplement: Supplementary file 1 [file ci6c00034_si_001.pdf]

Supplementary Information for

Improving AlphaFold2 performance in virtual screens targeting GPCRs by enhancing binding-site conformational sampling

Núria Mitjavila-Domènech[1,†], Alejandro Díaz-Holguín[1,†], Huabin Hu[1], Nour Aldin Kahlous[1], Israel Cabeza de Vaca[1], Björn Wallner[2,], Jens Carlsson[1,*]*

[1] Science for Life Laboratory, Department of Cell and Molecular Biology, Uppsala University, BMC Box 596, SE-751 24 Uppsala, Sweden

[2] Division of Bioinformatics, Department of Physics, Chemistry and Biology, Linköping University, 581 83 Linköping, Sweden

TABLE OF CONTENTS

Supplementary Tables

Table S1. Average TM6 Cα RMSD (Å) of AF2 and AF2-Multimer predictions to experimentally determined active and inactive receptor structures S3

Table S2. Summary of the available experimental structures for the 10 GPCRs........ S4

Table S3. Means of the binding-site volumes of experimental structures and AF2-based models........ S7

Table S4. Receptor G protein subunit type S8

Table S5. Number of ligands and property-matched decoys used in the docking calculations S9

Table S6. Maximum aLogAUC depending on AFsample2T ensemble size determined via bootstrapping S10

Table S7. Maximum aLogAUC depending on AF2 ensemble size determined via bootstrapping........ S11

Table S8. Ligand enrichment (EF1%) for the 10 GPCRs S12

Table S9. Definition of the extracellular TM region and part of EL2 that column masking was applied to S13

Supplementary Figures

Figure S1. AFsample2T binding-site models with 50% column masking........ S14

Figure S2. Structural variation of binding-site side chains........ S15

Figure S3. Structural variation of binding-site backbone S16

Figure S4. Relationship between calculated (AF2 and AFsample2T) and experimental per-residue binding-site RMSF values for 10 GPCRs........ S17

Figure S5. Relationship between calculated (AF2 and AFsample2T) and experimental per-residue binding-site RMSF values for D_1 and TAAR1........ S18

Figure S6. Comparison of models and experimental structures for 5-HT_{1A}, A_{2A}, D_1, D_2, and H_1 .... S19

Figure S7. Comparison of models and experimental structures for M_1, M_4, MT_1, μ-opioid, and TAAR1 .... S20

Figure S8. Ligand enrichment (aLogAUC) by AF2-based models and experimental structures S21

Figure S9. Ligand enrichment (aLogAUC) by the top 1% AF2-based models and experimental structures S22

Figure S10. Distribution of aLogAUC and EF1% values across 10 GPCRs........ S23

SUPPLEMENTARY TABLES

Table S1. Average TM6 Cα RMSD (Å) of AF2 and AF2-Multimer predictions to experimentally determined active and inactive receptor structures. The AF2-based prediction that is closest to the experimentally determined structures has been marked green. The AF2-Multimer predictions were performed using both the receptor and G protein sequences, whereas the AF predictions were performed using only the receptor sequence.

GPCR (residues in TM6)	RMSD to active structures (Å)		RMSD to inactive structures (Å)	
	AF2-Multimer	AF2	AF2-Multimer	AF2
D_2 (371-399)	1.03	1.38	1.28	0.73
H_1 (408-441)	0.68	1.21	0.98	0.69
M_1 (360-391)	0.60	1.73	1.47	0.57
M_4 (396-426)	0.31	0.75	1.01	0.58

Table S2. Summary of the available experimental structures for the 10 GPCRs.

GPCR	PDB CODE	METHOD	RESOLUTION (Å)	BOUND LIGAND (HA)[†]	STATE (A/I)[‡]	BINDING SITE MUTATIONS	USED FOR RMSD[§]
**Histamine H1 receptor (H1)** **AMINERGIC P35367**	3RZE[¦]	X-Ray	3.10	21	I	NO	YES
	7DFL	Cryo-EM	3.30	8	A	NO	YES
	8X5X	Cryo-EM	3.50	Apo	I	NO	YES
	8X5Y	Cryo-EM	3.00	34	I	NO	YES
	8X63	Cryo-EM	3.20	21	I	NO	YES
	8X64	Cryo-EM	3.40	22	I	NO	YES
**Adenosine receptor A2A (A2A)** **NUCLEOTIDE P29274**	2YDO[¦]	X-Ray	3.00	19	I	T65A, Q89A	NO
	4EIY[¦]	X-Ray	1.80	25	I	NO	NO
	5IU4[¦]	X-Ray	1.70	25	I	S253A	NO
	5IU7[¦]	X-Ray	1.90	30	I	S253A	NO
	5IU8[¦]	X-Ray	2.00	25	I	S253A	NO
	5IUA[¦]	X-Ray	2.20	31	I	S253A	NO
	5IUB[¦]	X-Ray	2.10	32	I	S253A	NO
	5K2C[¦]	X-Ray	1.90	25	I	NO	NO
	5K2D[¦]	X-Ray	1.90	13	I	NO	YES
	5MZJ[¦]	X-Ray	2.00	14	I	S253A	NO
	5MZP[¦]	X-Ray	2.10	14	I	S253A	NO
	5NM2[¦]	X-Ray	1.95	25	I	S253A	NO
	5OLG[¦]	X-Ray	1.87	25	I	S253A	NO
	5OLV[¦]	X-Ray	1.99	24	I	S253A	NO
	5OLZ[¦]	X-Ray	1.90	21	I	S253A	NO
	5OM1[¦]	X-Ray	2.10	21	I	S253A	NO
	5OM4[¦]	X-Ray	2.00	21	I	S253A	NO
	5VRA[¦]	X-Ray	2.35	25	I	NO	NO
	6GT3	X-Ray	2.00	12	I	S253A	NO
	6LPJ	X-Ray	1.80	25	I	NO	NO
	6LPK	X-Ray	1.80	25	I	NO	NO
	6LPL	X-Ray	2.00	25	I	NO	NO
	6PS7	X-Ray	1.85	25	I	NO	NO
	6WQA	X-Ray	2.00	25	I	NO	NO
	6ZDR	X-Ray	1.92	24	I	S253A	NO
	6ZDV	X-Ray	2.13	25	I	NO	YES
	7PX4	X-Ray	2.25	57	I	NO	YES
	8C9W	X-Ray	2.11	32	I	NO	NO
	8CIC	X-Ray	2.10	32	I	NO	NO
	8CU7	X-Ray	2.05	28	I	NO	YES
	8FYN	X-Ray	2.00	25	I	NO	NO
	8GNE	X-Ray	2.30	28	I	NO	YES
	8JWY	X-Ray	2.33	34	I	NO	NO
	8JWZ	X-Ray	2.37	32	I	NO	NO
	8PWN	X-Ray	2.40	13	I	NO	NO

GPCR	PDB ENTRY	METHOD	RESOLUTION (Å)	BOUND LIGAND (HA)[†]	STATE (A/I)[‡]	BINDING SITE MUTATIONS	USED FOR RMSD[§]
**5-hydroxytryptamine receptor 1A (5-HT$_{1A}$)**<br>**AMINERGIC**<br>**P08908**	7E2X	Cryo-EM	3.00	Apo	A	NO	YES
	7E2Y	Cryo-EM	3.00	13	A	NO	YES
	7E2Z	Cryo-EM	3.10	30	A	NO	YES
	8W8B	Cryo-EM	3.00	12	A	NO	YES
**Mu-type opioid receptor (μ-Opioid)**<br>**PEPTIDE**<br>**P35372**	7SBF	Cryo-EM	2.90	25	A	NO	NO
	7SCG	Cryo-EM	3.00	28	A	NO	YES
	7T2G	Cryo-EM	2.50	30	A	NO	YES
	7T2H	Cryo-EM	3.20	30	A	NO	YES
	7U2K	Cryo-EM	3.30	29	A	NO	NO[¶]
	7U2L	Cryo-EM	3.20	28	A	NO	YES
	8EF5	Cryo-EM	3.30	25	A	NO	YES
	8EF6	Cryo-EM	3.20	21	A	NO	YES
	8EFB	Cryo-EM	3.20	27	A	NO	YES
	8EFL	Cryo-EM	3.20	26	A	NO	YES
	8EFO	Cryo-EM	2.80	25	A	NO	YES
	8EFQ	Cryo-EM	3.30	13	A	NO	NO
	8F7Q	Cryo-EM	3.22	10	A	NO	YES
	8F7R	Cryo-EM	3.28	52	A	NO	YES
	8K9K	Cryo-EM	2.98	13	A	NO	YES
	8K9L	Cryo-EM	3.05	13	A	NO	NO
**D(1A) dopamine receptor (D$_1$)**<br>**AMINERGIC**<br>**P21728**	7CKW	Cryo-EM	3.22	21	A	NO	NO
	7CKY	Cryo-EM	3.20	28	A	NO	YES
	7CKZ	Cryo-EM	3.10	11	A	NO	NO
	7CRH	Cryo-EM	3.30	22	A	NO	YES
	7F0T	Cryo-EM	3.10	11	A	NO	NO
	7F1O	Cryo-EM	3.13	11	A	NO	NO
	7F1Z	Cryo-EM	3.46	11	A	NO	NO
	7JV5	Cryo-EM	3.00	20	A	NO	YES
	7JVP	Cryo-EM	2.90	22	A	NO	YES
	7JVQ	Cryo-EM	3.00	20	A	NO	YES
	7LJC	Cryo-EM	3.00	20	A	NO	NO
	7LJD	Cryo-EM	3.20	11	A	NO	NO
	7X2C	Cryo-EM	3.20	21	A	NO	YES
	7X2D	Cryo-EM	3.30	28	A	NO	YES
	7X2F	Cryo-EM	3.00	11	A	NO	YES
	8IRR	Cryo-EM	3.20	22	A	NO	YES
**Muscarinic acetylcholine receptor 1 (M$_1$)**<br>**AMINERGIC**<br>**P11229**	5CXV[¦]	X-Ray	2.70	26	I	NO	YES
	6OIJ	Cryo-EM	3.33	14	A	NO	YES
	6WJC	X-Ray	2.55	21	I	NO	YES
	6ZFZ	X-Ray	2.17	24	I	NO	YES
	6ZG4	X-Ray	2.33	26	I	NO	YES
	6ZG9	X-Ray	2.50	24	I	NO	YES

GPCR	PDB ENTRY	METHOD	RESOLUTION (Å)	BOUND LIGAND (HA)[†]	STATE (A/I)[‡]	BINDING SITE MUTATIONS	USED FOR RMSD[§]
**D(2) dopamine receptor (D_2)**<br><br>**AMINERGIC P21728**	6CM4	X-Ray	2.87	30	I	I122A	NO
	6LUQ	X-Ray	3.10	26	I	I122A	NO
	7DFP	X-Ray	3.10	29	I	S121K	NO
	7JVR	Cryo-EM	2.80	43	A	NO	YES
	8IRS	Cryo-EM	3.00	22	A	NO	YES
**Muscarinic acetylcholine receptor 4 (M_4)**<br><br>**AMINERGIC P08173**	5DSG[¦]	X-Ray	2.60	26	I	NO	YES
	6KP6	X-Ray	3.00	Apo	I	I187A	NO
	7TRK	Cryo-EM	2.80	14	A	NO	NO
	7TRP	Cryo-EM	2.40	14	A	NO	YES
	7TRQ	Cryo-EM	2.50	14	A	NO	NO
	7TRS	Cryo-EM	2.80	10	A	NO	YES
	7V68	Cryo-EM	3.40	14	A	NO	NO
	7V69	Cryo-EM	3.40	Apo	A	NO	YES
	8E9X	Cryo-EM	2.70	22	A	Y113C, A203G	NO
	8FX5	Cryo-EM	2.45	19	A	NO	YES
**Melatonin receptor type 1A (MT_1)**<br><br>**MELATONIN P48039**	6ME2	X-Ray	2.80	19	I	G104A, W251F	NO
	6ME3	X-Ray	2.90	23	I	G104A, W251F	NO
	6ME4	X-Ray	3.20	18	I	G104A, W251F	NO
	6ME5	X-Ray	3.20	18	I	G104A, W251F	NO
	6PS8	X-Ray	3.30	23	I	G104A, W251F	NO
	7DB6	Cryo-EM	3.30	19	A	NO	YES
	7VGY	Cryo-EM	3.10	18	A	NO	YES
	7VGZ	Cryo-EM	3.30	19	A	NO	NO
**Trace amine-associated receptor 1 (TAAR1)**<br><br>**AMINERGIC Q96RJ0**	8JLN	Cryo-EM	3.24	18	A	NO	YES
	8JLO	Cryo-EM	3.52	12	A	NO	NO
	8JLP	Cryo-EM	3.23	23	A	NO	YES
	8JLQ	Cryo-EM	2.84	21	A	NO	YES
	8JLR	Cryo-EM	3.00	24	A	NO	YES
	8JSO	Cryo-EM	3.40	10	A	NO	YES
	8UHB	Cryo-EM	3.35	16	A	F112W	NO
	8W87	Cryo-EM	2.80	11	A	NO	YES
	8W88	Cryo-EM	2.60	12	A	NO	YES
	8W89	Cryo-EM	3.00	9	A	NO	YES
	8W8A	Cryo-EM	2.80	16	A	NO	YES
	8WC8	Cryo-EM	2.90	10	A	NO	YES
	8WCA	Cryo-EM	3.48	9	A	NO	NO

[†] HA, heavy atoms.

[‡] The letters A and I denote structures annotated as active or inactive according to GPCRdb.

[§] For the RMSD calculations used to assess the accuracy of AF2-based models, experimental structures with a resolution better than 3.5 Å (3.0 Å for the A_{2A} receptor) were included, and structures with mutations in the orthosteric binding site were excluded. If the same ligand was bound in several structures, the structure with the highest resolution was selected.

[¦] Structures released prior to the AlphaFold2 training data cutoff (April 30, 2018)."

Table S3. Means of the binding-site volumes of experimental structures and AF2-based models. The mean values for the top 1% largest pocket volumes among the models are shown in parenthesis.

GPCR (Binding Site Residue[†])	AF2 Volume (Å³)	AFsample2T Volume (Å³)	Experimental Volume (Å³)
**5-HT$_{1A}$ (116)**	312.3 (361.5)	298.6 (494.9)	359.7
**A$_{2A}$ (181)**	116.3 (142.1)	118.1 (214.3)	82.3
**D$_1$ (103)**	205.1 (280.9)	209.9 (397.7)	253.9
**D$_2$ (114)**	292.8 (345.5)	289.4 (488.7)	322.8
**H$_1$ (107)**	275.6 (322.6)	287.1 (482.1)	317.3
**M$_1$ (105)**	220.1 (277.6)	227.8 (450.0)	257.5
**M$_4$ (112)**	103.8 (177.3)	124.9 (381.6)	168.5
**MT$_1$ (112)**	210.0 (278.0)	233.2 (317.4)	268.1
**µ-Opioid (149)**	274.4 (396.3)	305.5 (452.4)	313.9
**TAAR1 (103)**	81.0 (139.9)	85.5 (214.9)	216.7
**MEAN**	**209.1 (272.2)**	**218.0 (389.4)**	**256.1**

[†] Residue defining the location of the binding site in the SiteMap calculations.

Table S4. Receptor G protein subunit type. The sequences were obtained from the active structures in the PDB.

GPCR	G Protein subunit type	PDB Code
**5-HT$_{1A}$**	Gα_i	7E2Y
**A$_{2A}$**	Gα_s	6GDG
**D$_1$**	Gα_s	7JVP
**D$_2$**	Gα_i	7JVR
**H$_1$**	Gα_i	7DFL
**M$_1$**	Gα_i	6OIJ
**M$_4$**	Gα_i	7TRK
**MT$_1$**	Gα_i	7VGY
**μ-Opioid**	Gα_i	8EF5
**TAAR1**	Gα_s	8JLN

Table S5. Number of ligands and property-matched decoys used in the docking calculations.

GPCR	ACTIVES	DECOYS
**5-HT$_{1A}$**	166	8,618
**A$_{2A}$**	202	10,296
**D$_1$**	73	3,824
**D$_2$**	199	10,375
**H$_1$**	71	3,682
**M$_1$**	101	5,208
**M$_4$**	52	2,792
**MT$_1$**	129	6,282
**µ-Opioid**	54	2,580
**TAAR1**	128	5,785

Table S6. Maximum aLogAUC depending on AFsample2T ensemble size determined via bootstrapping (mean ± SD).

GPCR	Number of models				
	10	100	250	500	1,000
**5-HT$_{1A}$**	9.9 ± 1.6	12.4 ± 0.7	12.9 ± 0.5	13.2 ± 0.3	13.4
**A$_{2A}$**	14.9 ± 1.6	17.5 ± 1.1	18.4 ± 0.9	19.0 ± 0.7	19.8
**D$_1$**	9.4 ± 2.6	13.5 ± 2.0	15.4 ± 1.8	16.6 ± 1.2	17.4
**D$_2$**	5.8 ± 2.7	10.7 ± 2.3	12.4 ± 1.9	13.8 ± 1.4	14.8
**H$_1$**	7.7 ± 2.1	11.2 ± 1.5	12.4 ± 1.3	13.4 ± 1.1	14.3
**M$_1$**	6.9 ± 1.3	9.2 ± 0.8	9.8 ± 0.4	10.0 ± 0.1	10.1
**M$_4$**	4.9 ± 2.3	8.9 ± 1.7	10.2 ± 1.5	11.2 ± 1.1	11.9
**MT$_1$**	3.3 ± 1.6	5.8 ± 0.7	6.4 ± 0.6	6.8 ± 0.5	7.3
**µ-Opioid**	10.1 ± 1.8	13.1 ± 1.4	14.2 ± 1.3	15.3 ± 1.1	16.2
**TAAR1**	20.8 ± 2.9	26.4 ± 2.8	28.7 ± 2.6	30.4 ± 1.9	32.0
**MEAN**	**9.4**	**12.9**	**14.1**	**15.0**	**15.7**

Table S7. Maximum aLogAUC depending on AF2 ensemble size determined via bootstrapping (mean ± SD).

GPCR	Number of models				
	10	100	250	500	1,000
**5-HT$_{1A}$**	6.9 ± 1.2	9.1 ± 0.9	9.8 ± 0.7	10.2 ± 0.5	10.7
**A$_{2A}$**	16.2 ± 1.0	17.7 ± 0.5	18.2 ± 0.4	18.4 ± 0.2	18.5
**D$_1$**	6.2 ± 2.1	9.5 ± 2.4	11.3 ± 2.4	13.0 ± 1.8	14.4
**D$_2$**	5.6 ± 1.4	7.8 ± 0.9	8.5 ± 0.7	9.0 ± 0.6	9.4
**H$_1$**	8.1 ± 1.7	10.9 ± 1.0	11.6 ± 0.7	12.1 ± 0.3	12.2
**M$_1$**	7.5 ± 1.3	9.9 ± 1.3	10.9 ± 1.3	11.8 ± 1.1	12.9
**M$_4$**	3.7 ± 2.3	7.6 ± 1.6	9.0 ± 1.3	9.8 ± 0.7	10.3
**MT$_1$**	0.2 ± 0.9	1.8 ± 0.6	2.2 ± 0.3	2.4 ± 0.2	2.5
**µ-Opioid**	10.8 ± 1.6	13.2 ± 0.9	13.9 ± 0.8	14.4 ± 0.7	15.1
**TAAR1**	18.2 ± 1.6	20.7 ± 0.9	21.4 ± 0.7	21.9 ± 0.6	22.4
**MEAN**	**8.3**	**10.8**	**11.7**	**12.3**	**12.8**

Table S8. Ligand enrichment (EF1%) for the 10 GPCRs. For the AF2 and AFsample2T models, the EF1% values correspond to the top 1% performing models (minimum–maximum, with the median in parentheses). For the experimental structures, all the calculated EF1% values are included. The best median enrichments are highlighted in green.

GPCR	AF2	AFsample2T	Experimental
**5-HT$_{1A}$**	3.6 – 4.3 (3.7)	5.6 – 8.1 (6.2)	1.5 – 12.7 (3.6)
**A$_{2A}$**	15.2 – 17.2 (15.2)	14.2 – 16.2 (14.9)	4.1 – 18.7 (11.2)
**D$_1$**	3.6 – 5.3 (3.8)	7.5 – 9.8 (8.3)	0.0 – 7.5 (1.5)
**D$_2$**	6.0 – 7.2 (6.6)	10.4 – 14.4 (11.8)	2.3 – 15.9 (5.1)
**H$_1$**	7.9 – 11.8 (8.7)	7.9 – 10.1 (8.7)	0.0 – 21.2 (7.8)
**M$_1$**	9.1 – 11.2 (9.4)	7.7 – 10.8 (7.8)	0.0 – 4.7 (1.6)
**M$_4$**	5.0 – 6.7 (5.8)	5.8 – 9.4 (7.1)	0.0 – 9.8 (0.0)
**MT$_1$**	3.3 – 3.4 (3.3)	5.0 – 7.4 (5.8)	0.0 – 6.5 (2.4)
**µ-Opioid**	10.3 – 12.9 (10.3)	10.3 – 13.6 (10.3)	0.0 – 7.7 (5.1)
**TAAR1**	8.1 – 14.2 (8.2)	13.4 – 21.2 (15.1)	0.0 – 7.8 (2.7)
**MEAN**	**7.2 – 9.4 (7.5)**	**8.8 – 12.1 (9.6)**	**0.8 – 11.3 (4.1)**

Table S9. Definition of the extracellular TM region and part of EL2 that column masking was applied to.

GPCR	Residue Numbers
**5-HT$_{1A}$**	36-50, 84-100, 105-122, 163-174, 187-206, 355-371, 378-393
**A$_{2A}$**	1-20, 54-68, 74-90, 131-142, 166-188, 243-259, 266-281
**D$_1$**	23-37, 72-88, 92-109, 150-161, 186-205, 282-295, 309-324
**D$_2$**	34-48, 82-98, 103-120, 162-173, 182-200, 383-400, 404-419
**H$_1$**	28-41, 75-90, 95-113, 154-164, 180-201, 425-442, 449-461
**M$_1$**	21-39, 73-89, 94-111, 152-169, 178-199, 375-391, 396-411
**M$_4$**	32-46, 80-96, 102-118, 159-175, 185-206, 410-426, 431-446
**MT$_1$**	24-41, 75-91, 96-113, 154-160, 177-198, 248-261, 264-288
**μ-Opioid**	67-84, 118-131, 137-155, 196-205, 219-245, 292-304, 311-331
**TAAR1**	21-37, 71-84, 95-109, 150-160, 182-201, 261-277, 282-297

SUPPLEMENTARY FIGURES

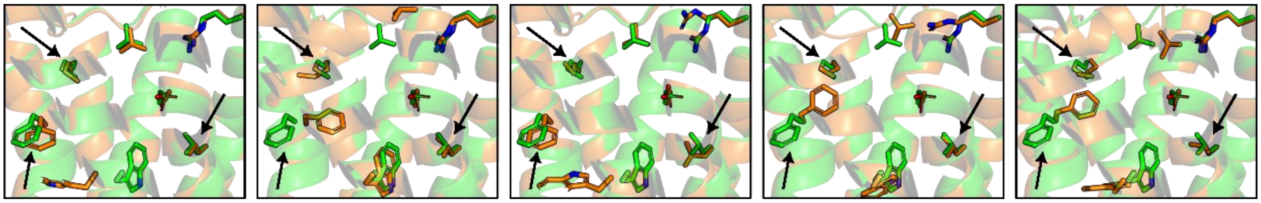

Figure S1. AFsample2T binding-site models with 50% column masking. AFsample2T predicted (orange cartoon) and experimentally (green cartoon) determined structures of TAAR1. Black arrows indicate three residues displaying large structural variation with 50% column masking. The binding-site RMSDs for the models are above 4 Å.

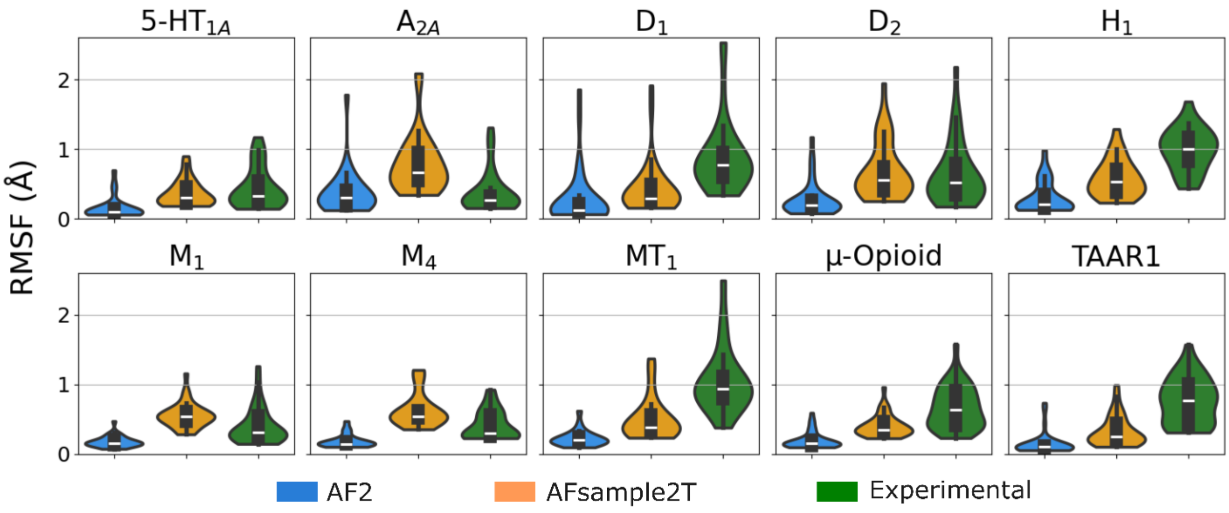

Figure S2. Structural variation of binding-site side chains. RMSF distributions of the side chains of binding-site residues across the 10 GPCRs: AF2 models (blue), AFsample2T ensembles (orange), and experimental structures (green).

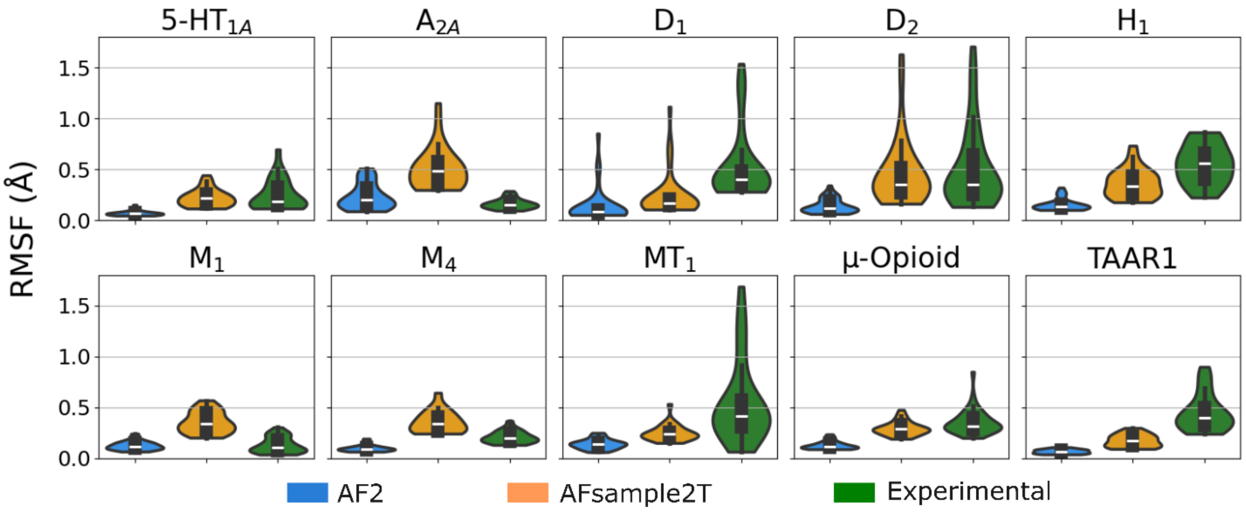

Figure S3. Structural variation of binding-site backbone. RMSF distributions of the backbone of binding-site residues across the 10 GPCRs: AF2 models (blue), AFsample2T ensembles (orange), and experimental structures (green).

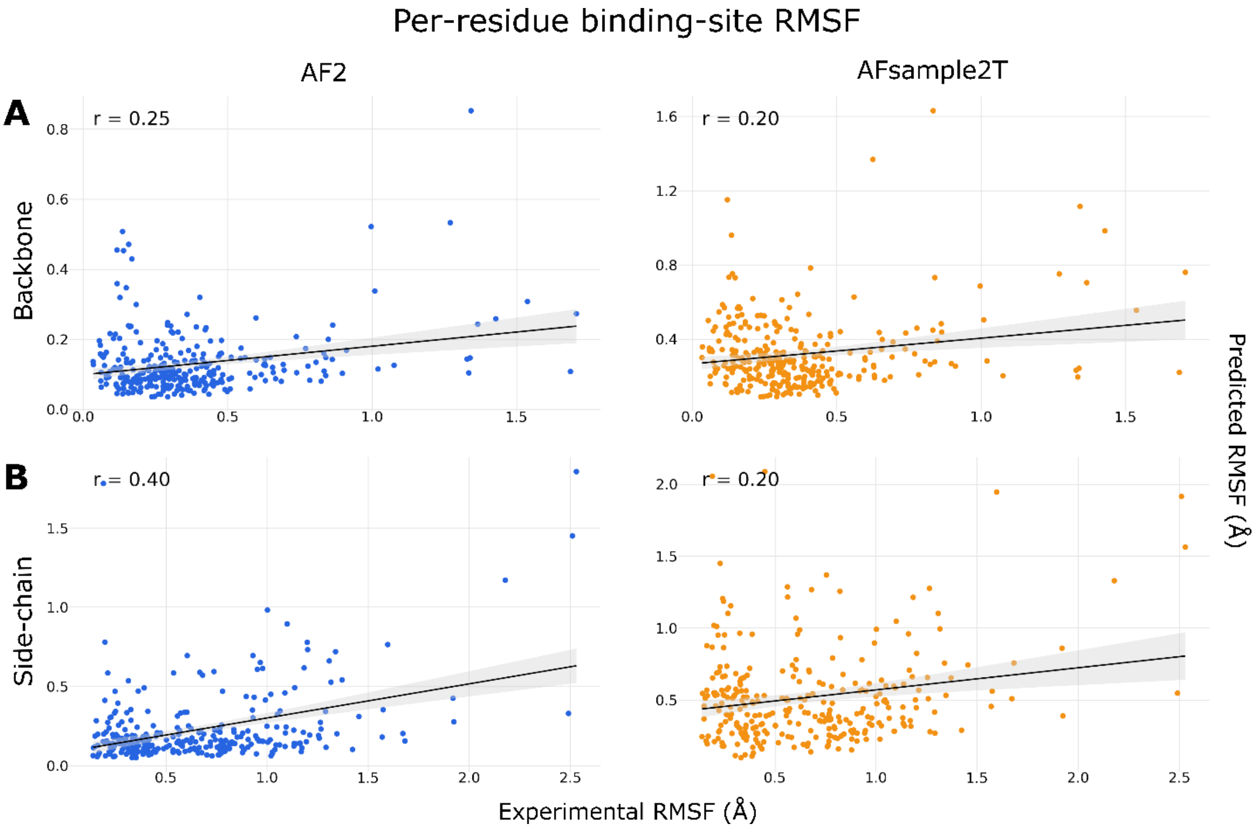

Figure S4. Relationship between calculated (AF2 and AFsample2T) and experimental per-residue binding-site RMSF values for 10 GPCRs. (A) Backbone and (B) side-chain atoms.

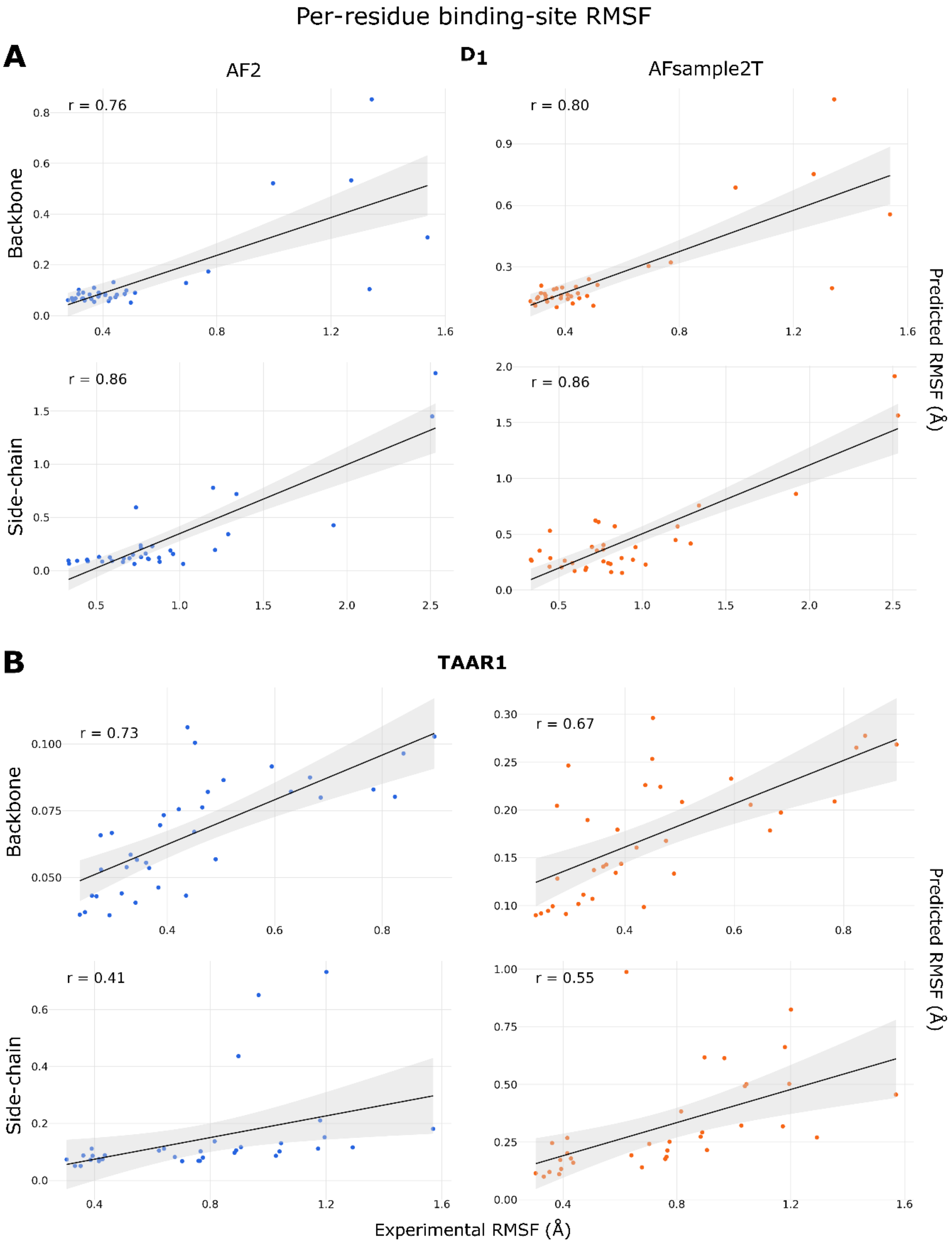

Figure S5. Relationship between calculated (AF2 and AFsample2T) and experimental per-residue binding-site RMSF values for D_1 and TAAR1. Backbone and side-chain atoms of (A) D_1 and (B) TAAR1.

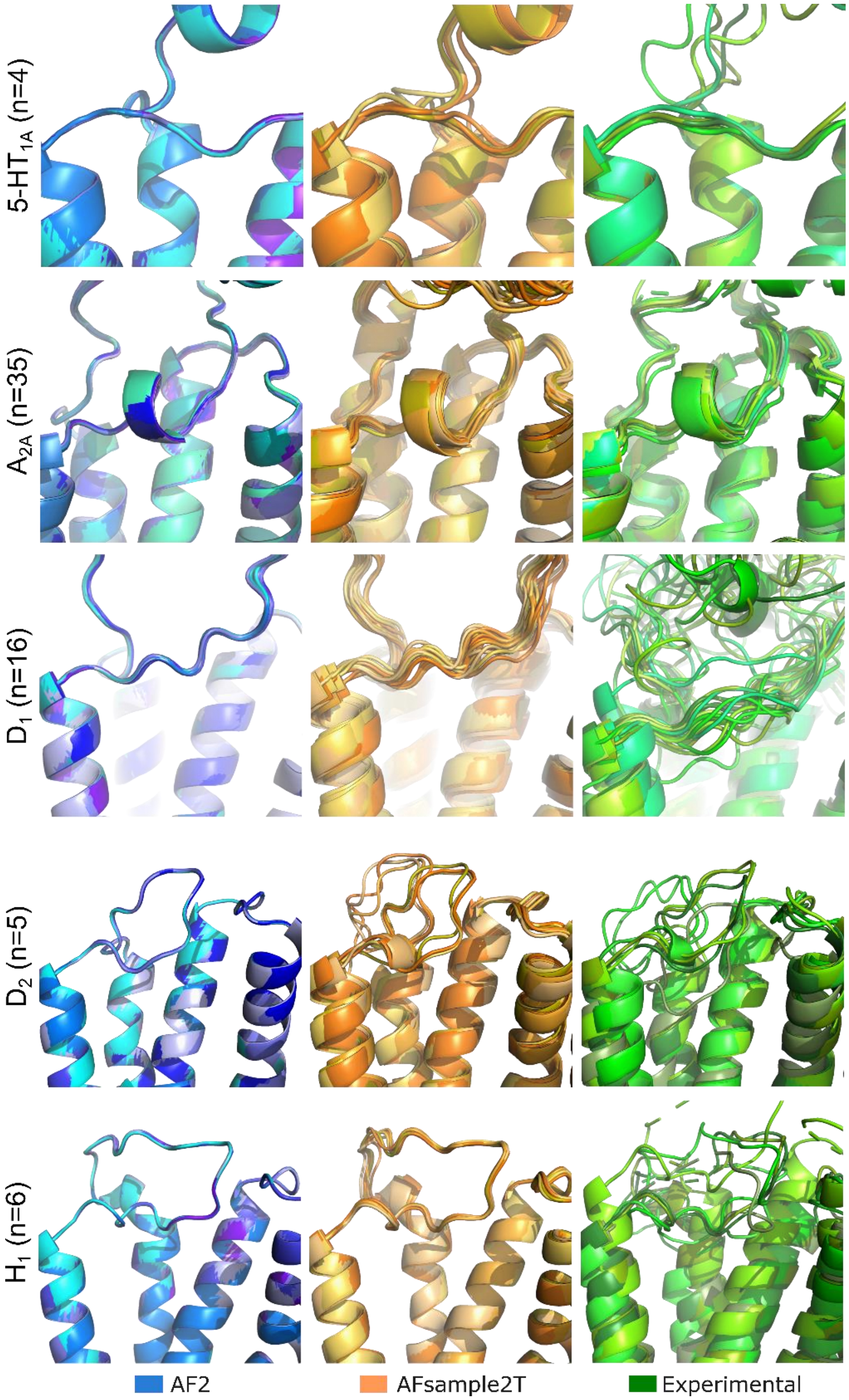

Figure S6. Comparison of models and experimental structures for 5-HT$_{1A}$, A$_{2A}$, D$_1$, D$_2$, and H$_1$. The EL2 region in AF2 models (blue) shows limited structural variability, whereas the AFsample2T ensemble (orange) and experimental structures (green) display greater and comparable variability.

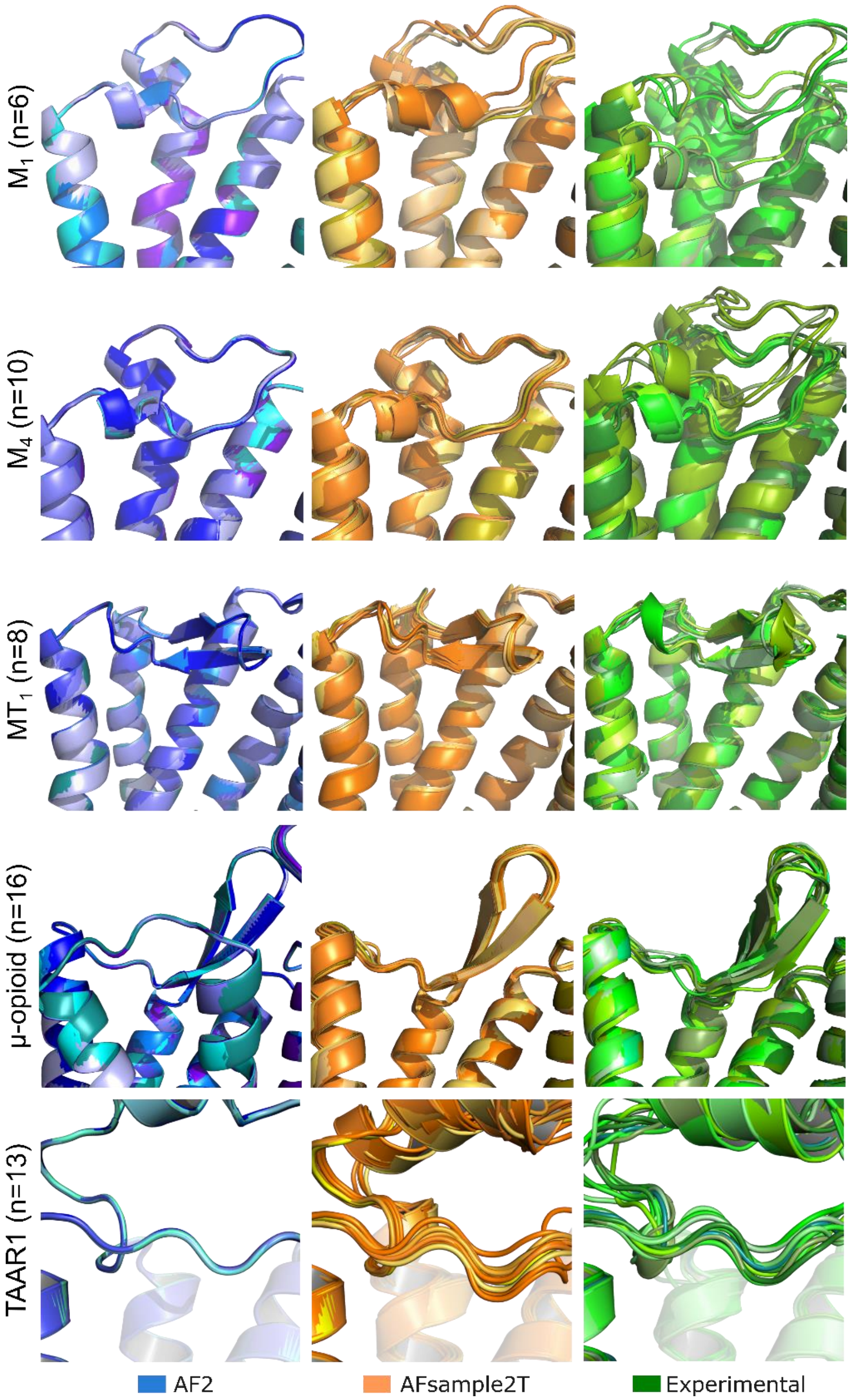

Figure S7. Comparison of models and experimental structures for M_1, M_4, MT_1, μ-opioid, and TAAR1. The EL2 region in AF2 models (blue) shows limited structural variability, whereas the AFsample2T ensemble (orange) and experimental structures (green) display greater and comparable variability.

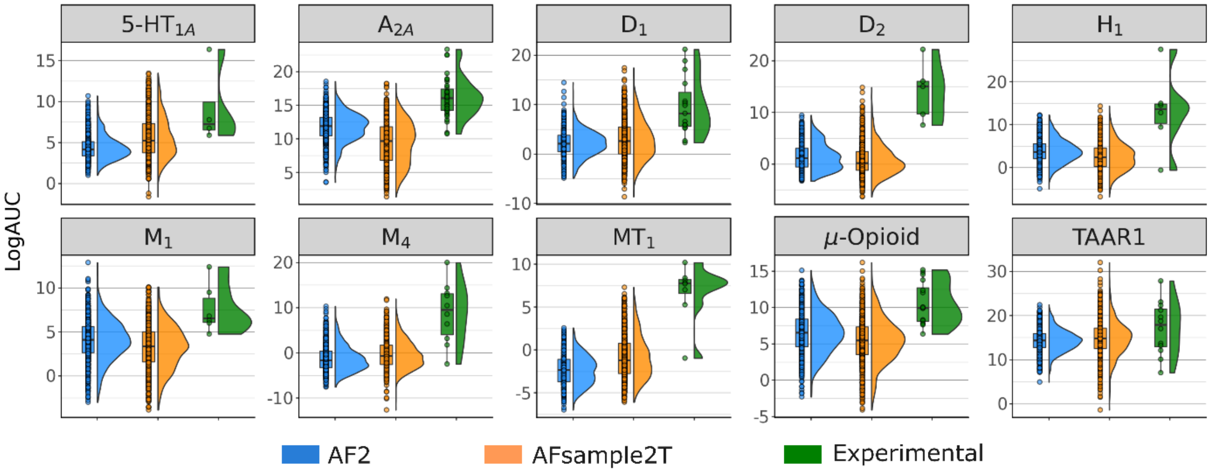

Figure S8. Ligand enrichment (aLogAUC) by AF2-based models and experimental structures. Violin plots of aLogAUC values from molecular docking calculations: AF2 models (blue, 1,000 models), AFsample2T ensembles (orange, 1,000 models), and experimental structures (green, 4-35 structures) for 10 GPCRs.

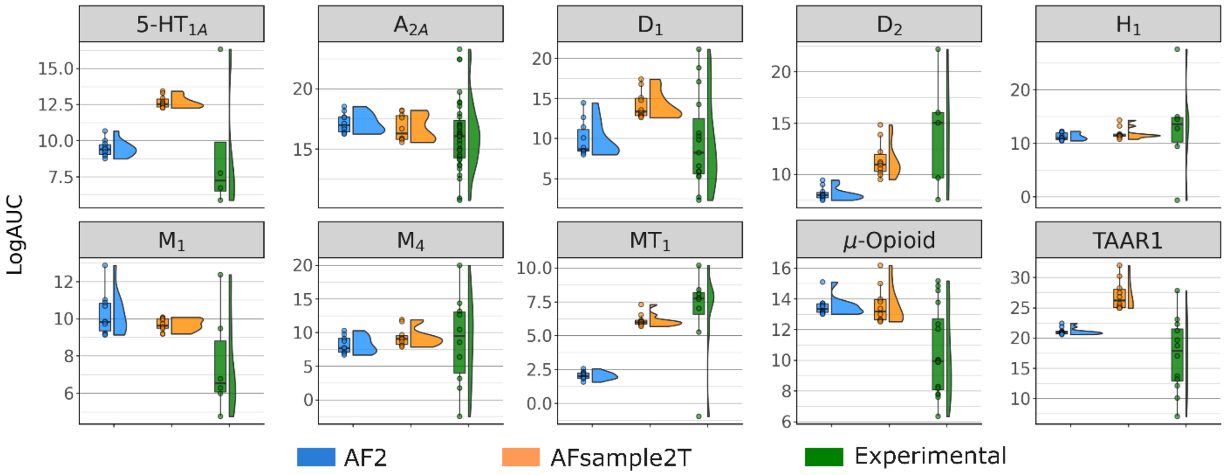

Figure S9. Ligand enrichment (aLogAUC) by the top 1% AF2-based models and experimental structures. Violin plots of aLogAUC values from molecular docking calculations: AF2 models (blue, 10 models), AFsample2T ensembles (orange, 10 models), and experimental structures (green, 4-35 structures) for 10 GPCRs.

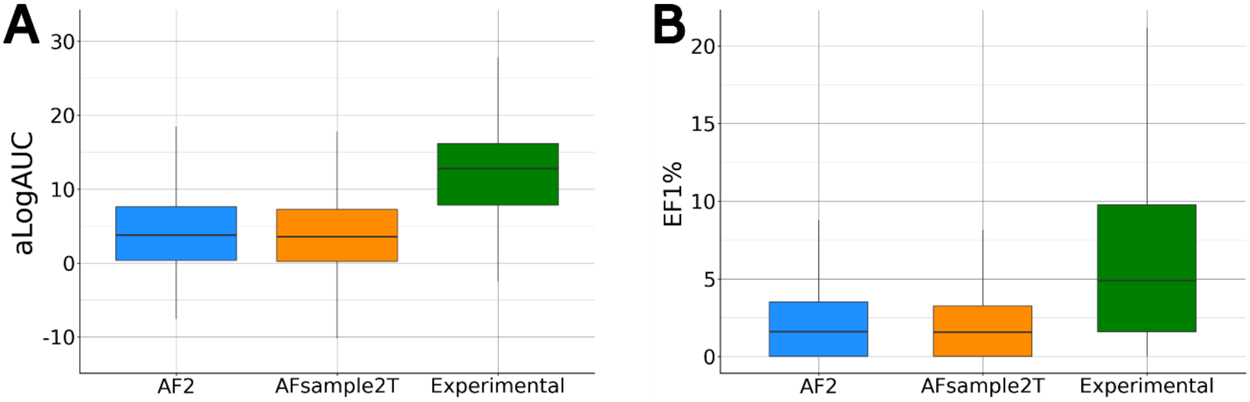

Figure S10. Distribution of aLogAUC and EF1% values across 10 GPCRs. Box plots of aLogAUC and EF1% values from molecular docking calculations: AF2 models (blue, 1,000 models per receptor), AFsample2T ensembles (orange, 1,000 models per receptor), and experimental structures (green, 4-35 structures) for 10 GPCRs.
